# A Proteomic Analysis of Nasopharyngeal Carcinoma in a Moroccan Subpopulation

**DOI:** 10.3390/cancers16193282

**Published:** 2024-09-26

**Authors:** Ayman Reffai, Michelle Hori, Ravali Adusumilli, Abel Bermudez, Abdelilah Bouzoubaa, Sharon Pitteri, Mohcine Bennani Mechita, Parag Mallick

**Affiliations:** 1 Intelligent Automation and BioMed Genomics Laboratory, Biology Department, Faculty of Sciences and Techniques of Tangier, Abdelmalek Essaadi University-Tetouan, Tangier 90000, Morocco; 2 Canary Center for Cancer Early Detection, School of Medicine, Stanford University, Stanford, CA 94305, USA; 3 Pathonord Pathology Laboratory, Tangier 90060, Morocco

**Keywords:** nasopharyngeal carcinoma, FFPE, proteomics, molecular profile, differentially expressed proteins, gene ontology, KEGG pathways, biomarkers

## Abstract

**Simple Summary:**

Nasopharyngeal carcinoma (NPC) is a head and neck cancer that is mainly found in Southeast Asia and North Africa. Most patients with NPC are diagnosed at an advanced stage, which increases the risk of recurrence and metastasis. A cohort of 41 NPC tumor samples from Morocco and North Africa were analyzed using shotgun proteomics. Within the cohort, three proteomically defined clusters were identified. We also examined sex and stage differences from a proteomic perspective. In total, 59 and 26 differentially abundant proteins were identified between male and female patients and patients with early and advanced stages within the cohort. Data were then compared to 21 healthy controls from a prior study. Across the two datasets, 6532 proteins were identified, of which 1507 proteins were differentially abundant between patients and controls. Differentially abundant proteins between tumor and healthy samples included PI3K, MAPK, BCL2, and PPIA proteins, which are involved in various biological pathways related to cell proliferation and growth. This study lays a foundation for both mechanistic and biomarker studies into NPC in Morocco and North African populations.

**Abstract:**

Background: Nasopharyngeal carcinoma (NPC) is a distinct cancer of the head and neck that is highly prevalent in Southeast Asia and North Africa. Though an extensive analysis of environmental and genetic contributors has been performed, very little is known about the proteome of this disease. A proteomic analysis of formalin-fixed paraffin-embedded (FFPE) tissues can provide valuable information on protein expression and molecular patterns for both increasing our understanding of the disease and for biomarker discovery. To date, very few NPC proteomic studies have been performed, and none focused on patients from Morocco and North Africa. Methods: Label-free Liquid Chromatography–Tandem Mass Spectrometry (LC-MS/MS) was used to perform a proteomic analysis of FFPE tissue samples from a cohort of 41 NPC tumor samples of Morocco and North Africa origins. The LC-MS/MS data from this cohort were analyzed alongside 21 healthy controls using MaxQuant 2.4.2.0. A differential expression analysis was performed using the MSstats package in R. Gene Ontology (GO) and Kyoto Encyclopedia of Genes and Genomes (KEGG) functional annotations were carried out using the DAVID bioinformatic tool. Results: 3341 proteins were identified across our NPC cases, revealing three main clusters and five DEPs with prognostic significance. The sex disparity of NPC was investigated from a proteomic perspective in which 59 DEPs were found between males and females, with significantly enriched terms associated with the immune response and gene expression. Furthermore, 26 DEPs were observed between patients with early and advanced stages of NPC with a significant cluster related to the immune response, implicating up-regulated DEPs such as IGHA, IGKC, and VAT1. Across both datasets, 6532 proteins were quantified between NPC patients and healthy controls. Among them, 1507 differentially expressed proteins (DEPs) were observed. GO and KEGG pathway analyses showed enriched terms of DEPs related to increased cellular activity, cell proliferation, and survival. PI3K and MAPK proteins as well as RAC1 BCL2 and PPIA were found to be overexpressed between cancer tissues and healthy controls. EBV infection was also one of the enriched pathways implicating its latent genes like LMP1 and LMP2 that activate several proteins and signaling pathways including NF-Kappa B, MAPK, and JAK-STAT pathways. Conclusion: Our findings unveil the proteomic landscape of NPC for the first time in the Moroccan population. These studies additionally may provide a foundation for identifying potential biomarkers. Further research is still needed to help develop tools for the early diagnosis and treatment of NPC in Moroccan and North African populations.

## 1. Introduction

Nasopharyngeal carcinoma (NPC) is a complex malignancy originating from the epithelial cells lining the nasopharynx, a region of the upper respiratory tract located behind the nasal cavity deep under the base of the skull. According to the most recent WHO classification published in 2022, NPC histological types are defined as keratinizing, non-keratinizing (undifferentiated and differentiated), and basaloid squamous cell carcinoma. Undifferentiated non-keratinizing carcinoma is the most common histologic subtype of NPC in endemic regions and is found to be strongly associated with Epstein–Barr virus (EBV) infection [1].

This type of cancer exhibits distinct epidemiological patterns and risk factors with a tendency for early local invasion and metastasis spread [2]. Its incidence is particularly high in Southeast Asia including the Cantonese population and in North Africa including Morocco, with age-standardized incidence rates (ASRs) of 2–10/100,000 per year. In other parts of the world, such as Northern America and Europe, NPC is rare with ASRs of less than 1 per 100,000/year according to the Global Cancer Observatory [3].

NPC shows a notable sex disparity with males being at high risk although few studies have investigated the causes of this remarkable difference [4]. In 2022, the incidence was estimated to be 120,434 new cases worldwide (males: 86,289; females: 34,145), and the mortality to be 73,482 worldwide (males: 54,104; females: 19,378) [3]. The significant mortality of the disease is partially attributed to its late presentation and diagnosis.

Nasopharyngeal carcinoma is a multifactorial disease, developed from a combination of genetic and environmental factors including the Epstein–Barr virus. The latter contributes to the oncogenesis of the nasopharynx by the expression of its latent genes such as the latent membrane protein (LMP1 and 2) and Epstein–Barr nuclear antigens (EBNA1 and 2) [5]. Apart from EBV and other environmental factors like diet and exposure to toxins, genetic predisposition and somatic alterations are found to play a crucial role in the development and progression of NPC. Oncogenes like Bcl-2 and tumor suppressor genes such as TGFBR2 as well as the dysregulation of signaling pathways including PI3K- AKT, MAPK, and NF-kB are reported to be implicated in NPC [6,7].

The tumor microenvironment (TME) is another characteristic of NPC that significantly contributes to NPC initiation, progression, and metastasis [8]. It is known for having heavy immune infiltration, involving several tumor-infiltrating immune cells, such as T lymphocytes, B lymphocytes, natural killer cells, macrophages, and other immune cells [9]. Other than being a multifactorial disease, NPC was recently proposed as an ecological and evolutionary disease, highlighting the role of TME in driving NPC’s aggressiveness, invasion, and metastasis [10].

Proteomics, the large-scale study of proteins, plays a crucial role in cancer research by enabling a better understanding of disease mechanisms and biomarker discovery. The identification of differentially expressed proteins and their association with biological and molecular pathways, driving cancer development and progression, provides valuable insights into disease mechanisms. Recent studies have shown the potential impact of proteomics studies of tumor tissues on identifying novel proteomically defined subtypes of disease and also the role of proteome alterations in initiation and progression [11,12]. Proteomics studies have also been critical for identifying both foundational mechanistic processes as well as identifying biomarkers for the early detection of cancers [13,14] and for potentially matching patients to targeted therapies [15]. Though proteomics technologies have been widely applied in cancer, there have been very few proteomics investigations of NPC. Xiao et al. analyzed the cellular proteome of NPC in the Chinese population, illuminating its differentially expressed proteins, including CTSD, KRT8, SFN, and STMN1, that were associated with NPC differentiation, suggesting their potential use as biomarkers for NPC prognoses [16]. Accordingly, Li et al. examined the proteome of NPC-derived cell lines, while Rong and colleagues investigated healthy and NPC tissues, as well as NPC-derived cell lines. These studies revealed the involvement of MAPK in NPC pathogenesis, progression, and radioresistance [17,18]. However, when it comes to studying nasopharyngeal carcinoma from a proteomic perspective, limited research has been performed in comparison with other types of cancer. Additionally, existing studies have focused dominantly on the Asian populations, specifically China. As of writing, we believe that this study represents the first investigation into the proteome of NPC in Morocco and the North African endemic region. The goal of our study has been to determine a foundational proteomic characterization of nasopharyngeal carcinoma in patients from the underserved, but critically important and prevalent, Morocco and North African region in order to better understand its molecular patterns. This baseline characterization may ultimately help identify biomarkers to improve the early diagnosis and treatment of this distinct head and neck cancer.

## 2. Materials and Methods

### 2.1. Sample Collection

Fifty-three formalin-fixed paraffin-embedded (FFPE) nasopharyngeal tissue blocks were obtained from the Pathonord pathology lab of Tangier at the time of diagnosis before any treatment was administered. These patients were diagnosed with nasopharyngeal carcinoma between 2017 and 2020 and followed up with at the Oncology Clinic and the Regional Center of Oncology of the University Hospital in Tangier, Morocco. All NPC cases were histologically confirmed to be undifferentiated non-keratinizing carcinoma according to the WHO classification. Additionally, the cancer cell area of the NPC tissues was confirmed by a pathologist before sample preparation at Stanford. The median age of all cases was 41 years old, with 66% being males and a sex ratio of 1.94 male/female. The TNM classification was provided for 22 patients with NPC according to the AJCC/UICC 8th edition with 90.91% being at an advanced stage (stages III, IVa-IVb). The present study was conducted in accordance with the Declaration of Helsinki and approved by the Ethics Committee for Biomedical Research (CERB 86-22). All participating patients provided informed consent. In addition, patients’ anonymity and confidentiality were ensured.

### 2.2. Sample Preparation

In total, 10 µm sections from the FFPE tissue blocks were sliced using a Thermo Microm HM355S Rotary Microtome Microtome (Thermo Fisher Scientific (Waltham, MA, USA)). Three sections per sample were then transferred into a microcentrifuge tube and kept on ice. Sample deparaffinization was performed by washing tissue once in xylene and twice in absolute ethanol with 10 min incubation periods and 2 min centrifugations at 15,000× *g*. After that, the samples were dried in a speed-vac for 10–15 min before adding the lysis buffer (100 mM Tris Hydrochloride—pH 8.0, 100 mM Dithiothreitol (DTT), 4% Sodium Dodecyl Sulfate (SDS), 1× Halt Protease Inhibitor Cocktail, and 1× Phenylmethylsulfonyl Fluoride (PMSF)) and vortexing. Briefly, samples were homogenized for 3 cycles of 1 min (1 cycle = 1 min ON, 1 min OFF) using the Pro Scientific Pro250 homogenizer (Oxford, CT, USA), sonicated at a 40% amplitude for 6 cycles (1 cycle = 30 s ON, 30 s OFF) using the Branson SLPe Sonifier (Danbury, CT, USA) while being kept on ice, and incubated in a thermos mixer at 600 rpm and 99 °C for 1 h. After centrifugation at 16,000× *g* for 10 min, proteins in the supernatant were transferred to a new tube while avoiding the pellet and quantified using a Thermo Micro-BCA assay kit (Waltham, MA, USA) after precipitating with acetone. In total, 41 samples had sufficient protein for a proteomics analysis.

Twenty-five micrograms of protein from each sample were precipitated with cold acetone (−20 °C), and incubated overnight at −20 °C. The following day, the mixture was centrifuged at 15,000× *g* for 15 min at 4 °C. The microcentrifuge tubes were then drained of cold acetone and refilled before being centrifuged again at 15,000× *g* for 10 min at 4 °C. This step was repeated before the pellet was air-dried for 30 min to 1 h. The protein pellet was resuspended afterward in 50 µL of 50 mM Ammonium Bicarbonate (ABC), followed by adding a 200 mM Tris(2-carboxyethyl)phosphine (TCEP) reducing agent and incubating for 1 h at 65 °C. The next steps of the tryptic protein digestion protocol included alkylating with 200 mM Iodoacetamide (IAA) in the dark for 30 min at room temperature, adding Trypsin 1:20, and incubating overnight at 37 °C. The labeled digests were air-dried for 45 min before reconstituting in 0.1% formic acid in LC-MS water, and transferred to autosampler vials for the LC-MS analysis.

### 2.3. LC-MS/MS Analysis

The reconstituted peptide samples were loaded onto a C18 trap column at a flow rate of 5 µL/min for 10 min after injecting 2 µL of the samples into a 10 µL loop using a Dionex Ultimate Rapid Separation Liquid Chromatography system from Thermo Fisher Scientific (Waltham, MA, USA). Tryptic peptides were separated using reversed-phase liquid chromatography, which involved two mobile phases, A and B. The first phase contained 0.1% formic acid in water, while the second had 0.1% formic acid in acetonitrile. The analytical column had a length of 25 cm and was packed with C18 BEH, 1.7 µm particle size (Waters Inc. (Milford, MA, USA)). The column was heated to 65 °C using a PST Phoenix S&T column heater (Research Triangle Park, NC, USA). The Orbitrap Eclipse Tribrid mass spectrometer from Thermo Fisher Scientific (Waltham, MA, USA) was used to analyze the eluted peptides by MS/MS. The gradient program was set to keep the mobile phase B at 2% during the initial 6 min, followed by a gradual ramp to 35% over the next 124 min, and then an increase to 85% over 5 min with a 5 min hold at a constant flow rate of 0.3 µL/min. The ion transfer tube temperature was set at 275 °C and the eluted peptides were ionized using the nano-spray (Thermo Fisher Scientific) at 2.2 kilovolts. The mass resolution was 240,000, and the scan ranged from 375 to 1800 *m*/*z*. For each MS1 scan, the radial frequency was set to 30%, with an ion intensity threshold of 5.0 e3 and a maximum injection time of 35 ms. The MS2 scans were with a cycle time of 1 ms in a Top-Speed mode. The dynamic exclusion time was set to a maximum of 30 s with a mass tolerance of 10 ppm, excluding isotopes. The maximum injection time for the MS2 automatic gain control (AGC) target was set at 50 ms. Using a mass isolation window of 0.7 *m*/*z*, the ion precursor underwent higher collisional dissociation (HCD) with a fixed collisional energy of 28% in the quadrupole. The turbo scan rate was used to detect the fragmented ions in the linear ion trap.

### 2.4. Bioinformatics and Data Analysis

#### 2.4.1. Protein Identification and Quantification

The LC-MS raw data were searched using MaxQuant (MQ) 2.4.2.0 software with the default parameters of the label-free quantification method [19]. It was performed in a single run, which includes 41 NPC samples (group 0 = disease) and 21 normal nasopharyngeal epithelial samples (group 1 = control). The raw data of the healthy individuals were obtained from the ProteomeXchange Consortium via the iProX partner repository under the Id IPX0001265000 [20]. MQ searches are performed using the Andromeda search engine integrated into MaxQuant with the Uniprot-SwissProt Human proteome database as a reference.

An unsupervised clustering analysis was used to determine clusters in our NPC cohort without predefined labels in order to identify protein expression and abundance patterns. To do so, the k-means clustering method and silhouette analysis were carried out in the R programming language. Silhouette plots were generated using the cluster and factoextra packages in R.

#### 2.4.2. Differential Expression Analysis

The differential expression analysis of the pre-processed data-dependent acquisition (DDA) data was performed using the MSstats package version 4.6.5 in the R programming language [21]. The protein groups and evidence files of MaxQuant, along with annotation tables, were used as input. The differentially expressed proteins (DEPs) were investigated in several conditions, including NPC cluster comparison (cluster 1 vs. 2, cluster 1 vs. 3, and cluster 2 vs. 3), NPC vs. controls, male vs. female patients, and patients with early vs. advanced stages of NPC. Briefly, the MaxQtoMSstatsFormat function was used to format the MQ output and remove any contaminants. The data processing was carried out by the dataProcess function, which included a logarithm transformation, a quantile normalization, and an imputation with the default summary method of Tukey’s median polish (TMP). This was followed by the differential analysis using the groupComparison function with a level of statistical significance set at *p* ≤ 0.05. Finally, R packages such as the ComplexHeatmap 2.14.0, VennDiagram 1.7.3, and ggplot 2 3.4.4 were used to generate the heatmap, Venn diagrams, volcano plots, and other visualizations.

#### 2.4.3. Pathway Analysis

The pathway analysis of the DEPs was conducted using the DAVID bioinformatics tool [22]. The functional annotation of Gene Ontology (GO) and Kyoto Encyclopedia of Genes and Genomes (KEGG) was performed to examine the enriched terms of biological process (BP), cellular component (CC), and molecular function (MF) as well as KEGG pathways with a *p*-value cutoff of 0.05 using the EASE (Expression Analysis Systematic Explorer) test. The latter employs a modified Fisher’s exact test to determine the significantly enriched terms in the submitted gene/protein list.

Additionally, a functional annotation cluster analysis was carried out to explore the different clusters of all significantly enriched functions and pathways, along with the DEPs involved. The threshold for statistical significance was set to *p* < 0.05. The ggplot 2 R package was used to generate visualizations for significantly enriched GO and KEGG pathways.

#### 2.4.4. Statistical Analysis

The statistical analysis was carried out in R to explore the prognostic significance of the most significant differentially expressed proteins using their LFQ intensities concerning various clinicopathological characteristics of NPC. The *t*-test statistical method was utilized to compare protein expression between patients with early and advanced stages of NPC, while the ANOVA test was used to examine the expression significance of DEPs across different Tumor (T) and Nodal (N) stages. Tukey’s test, a post hoc analysis, was used to determine which group means were different from each other after finding significance in the ANOVA test. The threshold for statistical significance was set to *p* < 0.05. Additionally, Box plots of DEPs were generated individually using the ggplot 2 R package.

## 3. Results

### 3.1. Proteomic Analysis

**Figure 1:**
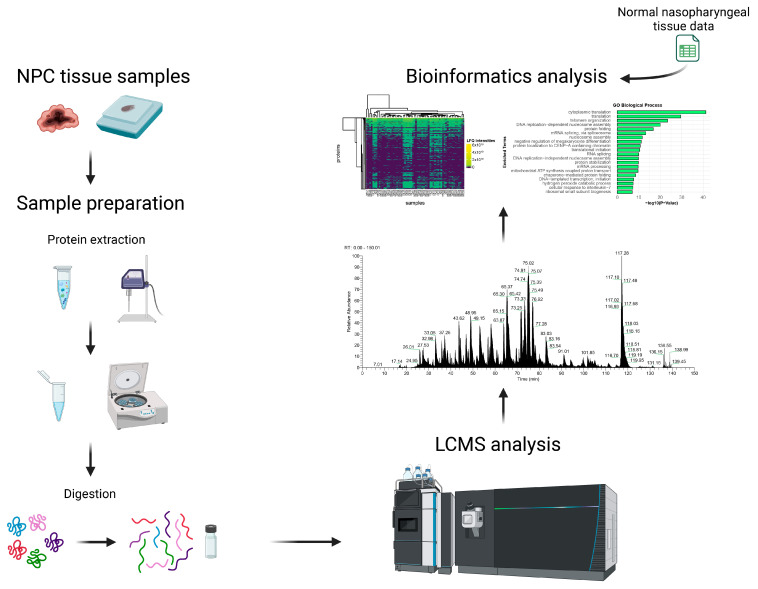
Label-free quantification shotgun proteomics workflow of nasopharyngeal carcinoma employed in this study.

**Figure 2:**
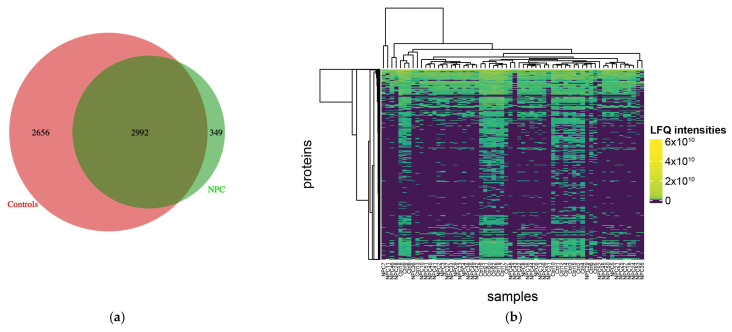
Protein identification and quantification in NPC and healthy control samples. (**a**): The Venn diagram showing the identified and overlapping proteins between NPC cases and healthy controls; (**b**): The heatmap of the label-free quantification protein abundances across all samples.

In this study, a label-free shotgun proteomics analysis was conducted on nasopharyngeal carcinoma (NPC) tissue from 41 patients of Morocco and North African origins as shown in Figure 1. In our collected dataset, 3341 proteins and 16,887 unique peptides were identified, ranging from 7 to 52 amino acids. These data were additionally jointly analyzed with data from 21 healthy control samples from Fu Ying et al. [20] Across all 62 samples, a total of 6532 proteins and 67,354 unique peptides were identified across both datasets, ranging from 7 to 52 amino acids (Supplemental Material Table S1). A Venn diagram was used to present the identified and overlapped proteins between the different sample sets (Figure 2a). The distribution of the proteins’ LFQ intensity values is shown in the heatmap plot (Figure 2b). As noted in the heatmap, there are several clusters of both cancer and healthy tissue proteomes.

### 3.2. Determination of Proteomically Defined Patient Groups

**Figure 3:**
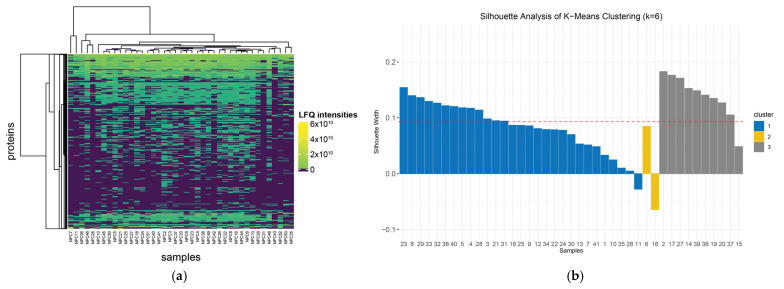
Protein quantification and clustering in NPC samples. (**a**) The heatmap of the label-free quantification protein abundances across all NPC samples. (**b**) The silhouette plot representing the 3 identified clusters.

The visual inspection of the heterogeneity of protein expression across patients and of the unsupervised clustering within the heatmaps suggested that there were potentially subtypes of NPC that were discernible from the proteomics data. To define the most likely clusters, we performed a k-means clustering analysis across a range of k from 1 to 7. The silhouette scores suggested that the most probable number of clusters was 3 (k = 3 had a silhouette score of 0.093). The first cluster contained 29 NPC samples, while the second and third contained 2 and 10 NPC samples, respectively. The distribution of proteins’ LFQ intensity in all NPC tissue samples and the identified clusters is represented in a heatmap and a silhouette plot (Figure 3a,b). We note that the silhouette score is low but significant and that cluster 2, in particular, had a low silhouette width.

**Figure 4:**
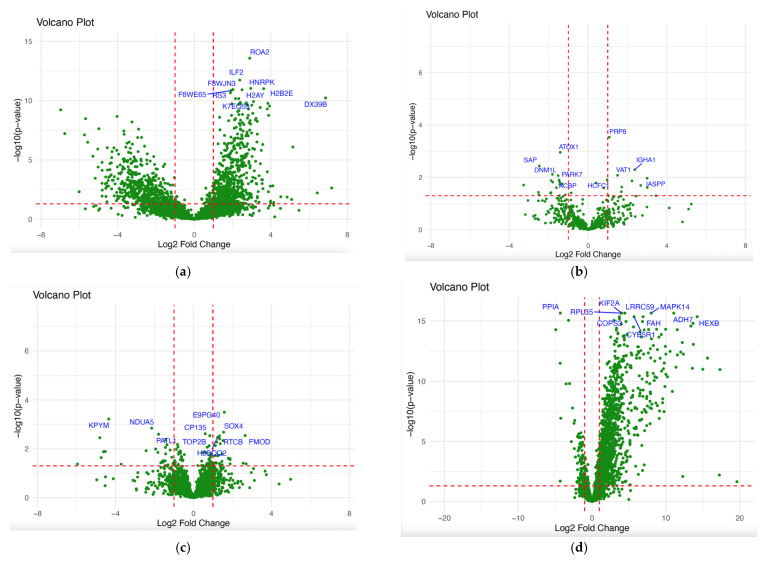
Volcano plots showing the differentially expressed proteins with an absolute 1 log2 fold change and *p*-value ≤ 0.05 among the following: (**a**). the NPC cluster condition (cluster 1 vs. 2, cluster 1 vs. 3, and cluster 2 vs. 3); (**b**). early versus advanced stage condition; (**c**). male versus female condition; and (**d**). NPC case versus control condition.

After identifying which patients belonged in each cluster, we then attempted to identify the proteins that differentiated amongst the clusters. The comparison between cluster 1 (*n* = 29) and cluster 3 (*n* = 10), being the more substantial comparison, showed 544 DEPs, with 468 up-regulated (fold change ≥ 1) and 76 down-regulated proteins (fold change ≤ 1) (Figure 4a, Table 1). The other comparisons involving cluster 2 (*n* = 2) can be found in Tables S2–S4 and Supplemental Material S5. The 10 most significant differentially expressed proteins (DEPs) across the NPC patient groups are found in Table 2. Among these differentially expressed proteins, the expression of HNRNPK and DAZAP1 proteins, identified between clusters 1 and 3, showed statistical significance across N stages (Nx-N0, Nx-N1, Nx-N2) (Supplemental Material S13).

The Gene Ontology (GO) enrichment analysis was conducted to obtain biological insights into three categories: the cellular component (CC), biological process (BP), and molecular function (MF). The significantly enriched terms of each category are presented in Supplemental Material S6 and Tables S7–S9. The analysis of inter-cluster differences between clusters 1 and 3 revealed BP-enriched terms including “cytoplasmic translation”, “translation”, “telomere organization”, “nucleosome assembly”, and “chromatin organization”. CC-enriched terms were involved in “extracellular exosome”, “cytosol”, “cytosolic ribosome”, “focal adhesion”, and “nucleus”. The DEPs in molecular function revealed enriched terms related to “RNA binding”, “protein binding”, “cadherin binding”, “structural constituent of chromatin”, and “structural constituent of ribosome”.

An examination of Kyoto Encyclopedia of Genes and Genomes Pathway (KEGG) enrichment analysis results (Figures S4–S6 in Supplemental Material S6, and Table S14) revealed that pathway enrichments in NPC cluster 1 versus 3 were related to “Ribosome”, “Coronavirus disease_COVID_19”, “Systemic lupus erythematosus”, “Neutrophil extracellular trap formation”, and “Alcoholism”.

### 3.3. Examination of Stage-Dependent Differences in NPC

**Figure 5:**
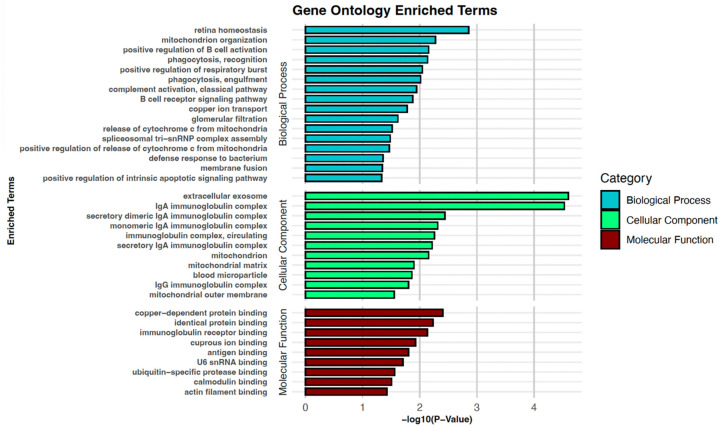
Gene Ontology enrichment analysis of DEPs observed between patients with early and advanced stages of NPC. BP-, CC-, and MF-enriched terms are presented.

The majority of patients with NPC are diagnosed at a late stage, resulting in a poorer prognosis. By identifying proteins that distinguish early-stage patients from late-stage patients, it may be possible to discern mechanisms of progression, identify biomarkers that may be used to stratify patients by stage, or ultimately even diagnose patients at their earliest and most treatable stages. For 22 of the patients in the cohort, NPC stage information was available; an analysis of proteins that differentiated patients with early NPC from patients with an advanced stage of NPC revealed 26 DEPs between patients, of which 9 were up-regulated and 17 were down-regulated proteins (Figure 4b, Table 1). Gene Ontology of the DEPs in the early versus advanced stage condition revealed significantly enriched terms of the biological process related to “retina homeostasis”, “mitochondrion organization”, “positive regulation of B cell activation”, “phagocytosis, recognition”, and “positive regulation of respiratory burst”. The main CC-enriched terms were “extracellular exosome”, “IgA immunoglobulin complex”, “secretory dimeric IgA immunoglobulin complex”, “monomeric IgA immunoglobulin complex”, and “immunoglobulin complex, circulating”. Finally, the significantly enriched terms of MF were related to binding processes like “copper-dependent protein binding”, identical protein binding”, “immunoglobulin receptor binding”, “cuprous ion binding”, and “antigen-binding” (Figure 5, and Table S16).

### 3.4. Examination of Sex-Dependent Differences in NPC

**Figure 6:**
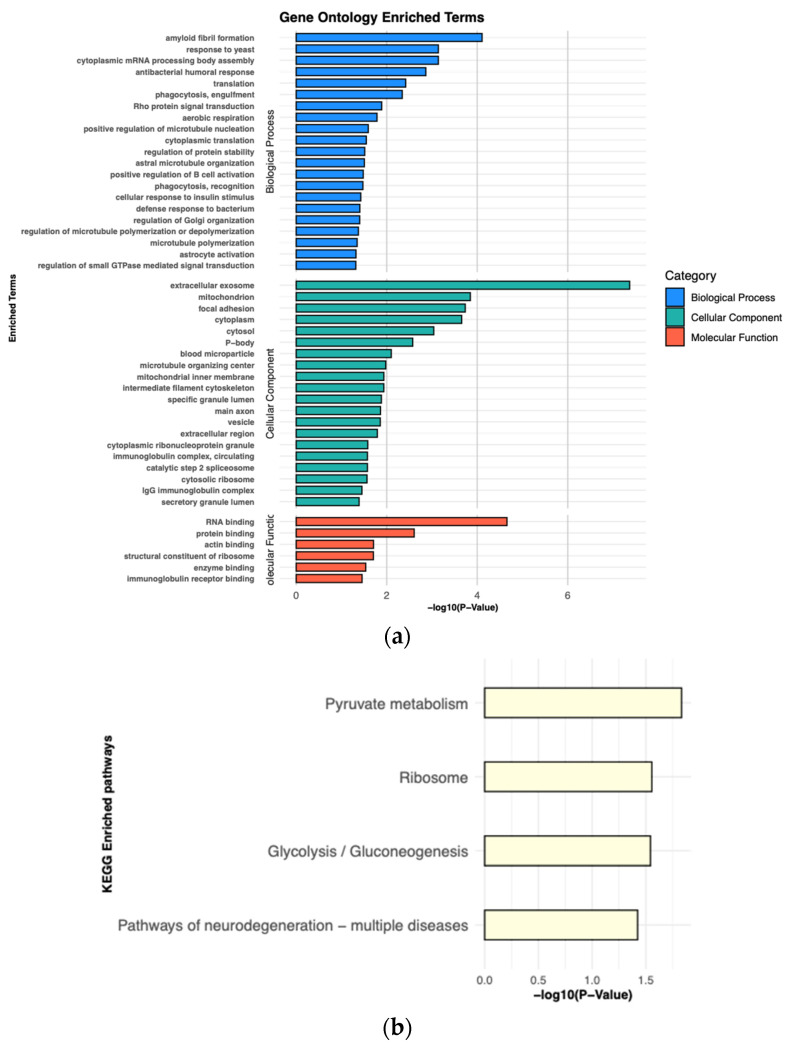
Pathway analysis of DEPs observed between male and female patients. (**a**). Gene Ontology enrichment analysis. Enriched terms of biological process, cellular component, and molecular function are presented. (**b**). Enriched terms of KEGG pathway analysis are presented.

Given the strong sex-dependent differences in the incidence and prognosis, we additionally examined our cohort to identify proteins that distinguished tumors derived from male vs. female patients (Figure 4c). A total of 59 proteins were identified as differentially expressed between males and females of NPC samples, with 30 up-regulated and 29 down-regulated proteins. When comparing male and female patients with NPC, it was found that the main enriched terms of BP were “amyloid fibril formation”, “response to yeast”, “cytoplasmic mRNA processing body assembly”, “antibacterial humoral response”, and “translation”. Meanwhile, DEPs in CC were mostly involved in “extracellular exosome”, “mitochondrion”, “focal adhesion”, “cytoplasm”, and “cytosol”. The MF-enriched terms were mostly involved in binding processes, including “RNA binding”, “protein binding”, “actin binding”, “structural constituent of ribosome”, and “enzyme binding” (Figure 6a, and Table S15). The pathways enriched in KEGG included “Pyruvate metabolism”, “Ribosome”, “Glycolysis/Gluconeogenesis”, and “Pathways of neurodegeneration multiple diseases” (Figure 6b, and Table S18).

### 3.5. Examination of Tumor-Specific Differences in Proteomics Profiles

By leveraging the joint analysis of our data alongside healthy control data, it was possible to identify proteins that potentially distinguish healthy tissue from NPC tissue. The results of the differential expression analysis for the identified proteins using a *p*-value ≤ 0.05 can be seen in Figure 4d and Table 1. A total of 1507 DEPs were observed between NPC and control samples, with 1408 up-regulated and 99 down-regulated proteins. The list of identified proteins and DEPs for all conditions can be found in Supplementary Tables S1–S4 and S10–S12. The top 10 largest DEPs for NPC vs. control conditions are presented in Table 3. As expected, housekeeping proteins, including actins (ACTA, ACTB, ACTG), and beta-2-microglobulin (B2M) did not change in the differential expression analysis (negative control proteins), suggesting that there were not systematic biases in the data. Though anticipated to be associated with NPC, JNK proteins (MAPK8, MAPK9, MAPK10), tumor suppressor ARF (CDKN2A), Ras association domain-containing protein 1 (RASSF1), and cyclin D1 (CCND1) were not observed as statistically significant DEPs. We additionally investigated which DEPs may have been associated with the tumor stage, finding that PPIA’s expression showed statistical significance across different T stages, particularly between T2 and T3. The results of this investigation are presented in Supplementary Material S13.

**Figure 7:**
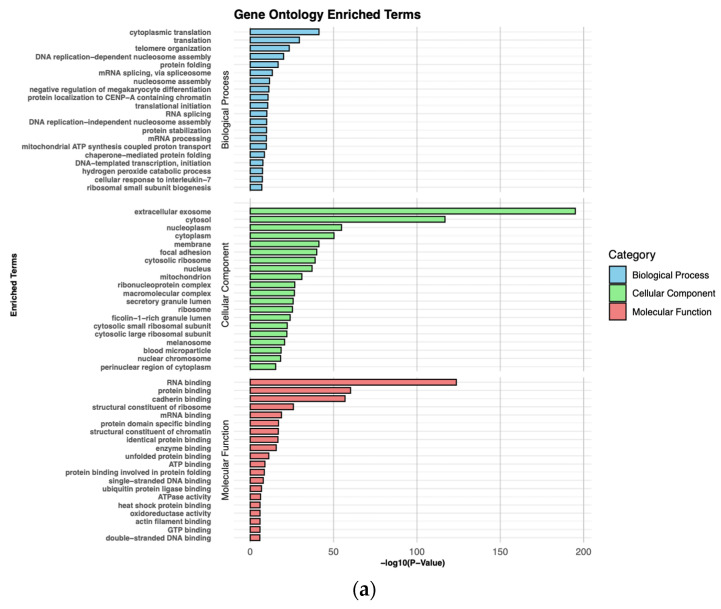

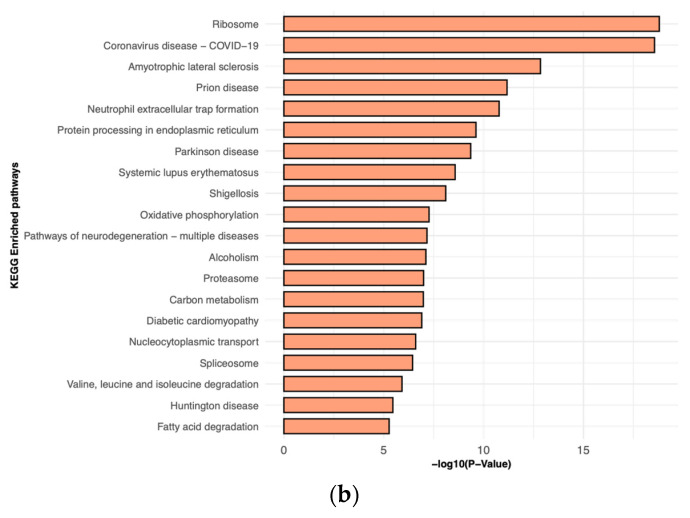
Pathway analysis of DEPs observed between NPC cases and healthy controls. (**a**). Gene Ontology enrichment analysis. Twenty most significant enriched terms of biological process, cellular component, and molecular function are presented. (**b**). KEGG pathway analysis. Twenty most significant enriched pathways are presented.

In NPC cases versus controls, the main enriched BP terms were involved in “cytoplasmic translation”, “translation”, “telomere organization”, “DNA replication-dependent nucleosome assembly”, and “protein folding”. Most of the differentially expressed proteins related to CC were associated with “extracellular exosome”, “cytosol”, “nucleoplasm”, “cytoplasm”, and “membrane”. Regarding MF, the main enriched terms were related to “RNA binding”, “protein binding”, “cadherin binding”, “structural constituent of ribosome”, and “mRNA binding” (Figure 7a and Table S17). The main enriched KEGG pathways in NPC vs. control involved differentially expressed proteins in “Ribosome”, “Coronavirus disease_COVID_19”, “Amyotrophic lateral sclerosis”, “Prion disease”, and “Neutrophil extracellular trap formation” (Figure 7b and Table S19).

## 4. Discussion

In this study, we employed a high-throughput shotgun proteomics approach that involved label-free quantification LC-MS/MS and data-dependent acquisition on FFPE tissue samples from patients with nasopharyngeal carcinoma. To the best of our knowledge, this is the first study of its kind on the Moroccan population and the North African region using a proteomic approach, and one of the few studies worldwide identifying the proteomic profile of this complex disease via FFPE tissues. We performed a bioinformatics analysis of label-free shotgun proteomics data from forty-one samples of nasopharyngeal carcinoma tissue collected herein. Within our dataset, 3341 proteins and 16,887 unique peptides were identified.

Using our NPC dataset, we attempted to identify and characterize proteomically defined subtypes. A pathway analysis was performed on the identified DEPs across the three cluster comparisons to elucidate their biological significance in NPC. Although the comparison between clusters 1 and 3 was more substantial given their higher sample size, we retained the other comparisons providing valuable insights. The Gene Ontology analysis for cluster 1 compared to cluster 3 revealed significant enrichment in biological processes related to DNA/RNA and protein processing and stabilization as well as gene expression and translation processes, indicating increased cellular activity. A functional annotation clustering analysis, combining BP, CC, and MF from Gene Ontology and KEGG pathway results, was conducted to confirm these findings further. In the comparison between clusters 1 and 3, the most significant functional annotation cluster was related to the translation process and ribosomes.

In both cluster 1 compared to 3 and cluster 2 compared to 3, 7 out of the 10 most significant differentially expressed proteins were the same. All of these proteins were up-regulated in cluster 1 vs. 3 and down-regulated in cluster 2 vs. 3. In terms of prognostic significance, HNRNPK and DAZAP1 up-regulated proteins were both found to be significantly expressed across N stages between clusters 1 and 3. Several studies have shown the implication of the HNRNPK protein in cancer by interacting with other proteins and signaling pathways such as the WNT pathway [23]. Chung et al. found that HNRNPK’s high expression was correlated with the overexpression of MMP12 in NPC tissues, suggesting a potential therapeutic role for this protein [24]. Although DAZAP1 was less reported, it was shown to be involved in cell proliferation with its important role in m-RNA splicing activation [25]. Deng et al. found that the overexpression of DAZAP1 in hepatic carcinoma was a predictor of a poor prognosis [26]. These proteins, found to have prognostic significance in our study, might serve as novel markers, specifically in the early stages of NPC.

Nasopharyngeal carcinoma poses a significant challenge due to its late declaration problem, wherein most people are diagnosed at an advanced stage, resulting in a poorer prognosis. In this study, we aimed to analyze NPC stages from a proteomics perspective by investigating the differentially expressed proteins in this condition. A total of 26 DEPs were observed between the early and advanced stages of NPC.

Significantly enriched terms from Gene Ontology BP, CC, and MF showed a cluster related to the immune response. This could imply a strong immune reaction against tumor cells during the progression of NPC in the early stages. There may also be changes in the tumor microenvironment (TME), where variations in immune cells’ activity could impact the disease stage. These changes may enable cancer cells to evade detection and elimination by the immune system. It was previously reported that EBV-positive NPC TME was extensively infiltrated with immune cells such as T cells, B cells, natural killers, dendritic cells, macrophages, and other immune cells [9]. Several genes linked to NPC have been found to be involved in immune responses, such as the human leukocyte antigen (HLA) polymorphism [27]. Moreover, Wang et al. have found that E2F3, a transcription factor, induces an immunosuppressive TME in NPC by activating two proteins, namely RPC1 and BIRC5 [28]. A recent study suggested the involvement of EBV Rta, specifically BRLF1, in PD-L1 activation [29]. The latter, an important immune checkpoint target, may play a crucial role in modulating the tumor microenvironment of NPC.

The implicated DEPs in the significant cluster such as IGHA1 and IGHA2 (Immunoglobulin Alpha Chains), constant regions of immunoglobulin heavy chains and a component of IgA, and IGKC (Immunoglobulin Kappa Constant), a constant region of immunoglobulin light chains, were overexpressed, which further confirms the increased immune response. IGKC expression was found to be significantly correlated with a lower T stage and improved survival [30] and was reported as a strong immune marker in human cancer, predictive of favorable treatment response [31]. In fact, high immune activity in the early stages of NPC might correlate with a better prognosis, showing the importance of early detection. Further interesting DEPs are discussed in Supplemental Material S20.

Besides the immune response, other up-regulated differentially expressed proteins (DEPs), such as pre-mRNA-processing-splicing factor 8 (PRP8), which plays a role in pre-mRNA splicing, were found to be of interest. Cao et al. found that PRP8 was a significant factor for splicing in the progression of breast cancer, being correlated with JAK-STAT, TGF-β, and cell cycle control pathways [32]. Meanwhile, Blazquez-Encinas et al. reported the implication of PRP8 in cell proliferation and colony formation in lung carcinoids [33]. Nevertheless, it is unclear how PRP8 is involved in NPC. It is also interesting to note that the BH3-interacting domain death agonist (BID), which plays an important role in apoptosis, was found to be down-regulated in the early versus advanced NPC stage condition. BID is a proapoptotic protein that counters the effect of the antiapoptotic BCL-2, which was found to be overexpressed in our study. Evidence has shown that BID expression was decreased in various cancer types, including hepatocellular carcinoma [34] and breast cancer [35], which further supports our findings. Understanding these potential biomarkers would help improve early diagnoses and provide insights into potential therapeutic targets including enhancing the immune response in the early stage.

Nasopharyngeal carcinoma shows a notable sex disparity in the incidence and prognosis. Various studies have highlighted this remarkable difference, with males being at higher risk although without knowing the exact cause [4]. Xie and colleagues also confirmed the male predominance and suggested a potential protective effect of estrogen on the risk of developing NPC based on the age-dependent sex ratio [36]. Hormones are known to play a role in modulating inflammation. In fact, estrogen was found to regulate the inflammatory response by activating or inhibiting the NLRP_3_ inflammasome that can either promote or ameliorate various diseases [37]. Besides incidence, Cui et al. reported a better prognosis in women compared to men with NPC, indicating a need for a more personalized therapeutic approach for this type of cancer [38].

In this study, we examined the protein expression in NPC using the sex difference condition for the first time. A total of 59 differentially expressed proteins were identified in males’ compared to females’ nasopharyngeal carcinoma tissue samples. Similar to the NPC versus control condition, the pathway analysis of the male versus female condition showed significant clusters of Gene Ontology and KEGG pathways related to gene expression, suggesting an increased cellular activity and growth. Interestingly, other significant clusters of DEPs were associated with the immune response, particularly the innate immune response, which might indicate an inflammatory response related to cancer. Inflammation can promote cancer development and progression and has been shown to play a role in response to therapy with the implication of several signaling pathways including NF-kB, JAK-STAT, and MAPK [39]. These findings might partly explain the association between inflammation, carcinogenesis, and the sex disparity in patients with nasopharyngeal carcinoma although it needs further investigation. This could be added to the greater exposure of men to NPC’s toxic and occupational risk factors such as chemical fumes and wood dust [40].

To complement our study of NPC, we identified samples of a normal nasopharynx from Fu Ying et al.’s paper [20]. We downloaded their raw data from the ProteomeXchange and processed the complete set of data together. We next examined the data to identify proteins with differential expression between disease and control populations. In total, 6532 proteins and 67,354 unique peptides were observed, out of which 1507 were differentially expressed in NPC cases versus healthy controls.

GO and KEGG pathway enrichment analyses were utilized to highlight all the differentially expressed proteins and determine their functional significance to nasopharyngeal carcinoma. The results of Gene Ontology annotation in NPC cases compared to controls revealed a significant enrichment in biological process terms related to DNA transcription, translation, and replication processes. Other terms were associated with protein folding and ATP synthesis. Moreover, the molecular function’s significantly enriched terms were related to binding functions, including RNA and protein binding, alongside terms related to structural constituents of ribosomes, chromatin, and ATPase activity, indicating an implication in gene expression and regulation as well as energy-dependent processes.

The functional annotation clustering analysis using the DAVID bioinformatics tool combining BP, CC, and MF of GO and KEGG pathways also showed significant clusters of DEPs associated with the transcription and translation processes, as well as DNA replication. This suggests an increased cellular activity, which might indicate enhanced cell proliferation. In addition, the DEPs associated with these processes were up-regulated, which might explain an overexpression leading to uncontrolled cell growth and proliferation, a known hallmark feature of carcinogenesis.

The following DEPs were highly enriched in the significant KEGG pathways: PI3KCA, PIK3R1, RAC1, BCL2, MAPK3 (ERK4), and p38 MAPK. Numerous studies have demonstrated that phosphatidylinositol 3-kinase (PI3K) genes, particularly PIK3CA and PIK3R1, have an oncogenic effect and are involved in various types of human cancers [41,42,43]. PIK3CA alteration has also been reported in nasopharyngeal carcinoma. Fendri et al. found that PIK3CA amplification predicted a poor prognosis in Tunisian patients with NPC [44]. In another study, Yip and colleagues showed that PIK3CA gene amplification may contribute to NPC pathogenesis along with the high expression of p110α [45]. These genes activate the PI3K-AKT signaling pathway, which plays a crucial role in cancer cell proliferation, survival, angiogenesis, and metastasis. As a result, PI3K inhibitors have been developed as therapeutic drugs [46], which could be useful for treating NPC. Similarly, RAC1 was found to be implicated in the regulation of cell migration and metastasis of several cancer types [47,48]. A study conducted by Yan Qi et al. found that RAC1 was overexpressed and significantly associated with the stage and grading of NPC [49], suggesting a role in NPC progression. In our study, RAC1 was also found to be up-regulated, which could serve as a potential prognostic biomarker. Evidence has shown that BCL2 is implicated in carcinogenesis by inhibiting apoptosis, thereby promoting cell survival [50]. Some studies also reported the overexpression of BCL2 in nasopharyngeal carcinoma, which may play an important role in lymph node metastasis [51]. Its up-regulation may represent a valuable prognostic factor for this type of cancer.

In this study, the MAPK3, also known as extracellular signal-regulated kinase 1 (ERK1), and p38 MAPK proteins such as MAPK11 (p38β) and MAPK14 (p38α) of the mitogen-activated protein kinase family were up-regulated. It was also observed that MAPK14 was the second most significant DEP. These MAPK proteins have been found to play a role in regulating cell cycle progression, survival, differentiation, metastasis, stress response, and inflammation [52]. In a study conducted by Farhat et al. in 2018, there was a significant correlation between p38 MAPK expression and the clinical stage of nasopharyngeal carcinoma [53]. Due to the important roles of MAPK family genes in NPC pathogenesis and other cancers, inhibitors of p38 MAPK and JNK have been developed and are currently undergoing clinical trials in cancer patients [54]. Further research is warranted to study the potential use of these proteins as molecular biomarkers for NPC.

In addition to the 20 most enriched KEGG pathways presented in Figure 7b, a noteworthy and significant pathway of interest observed was the Epstein–Barr virus (EBV) infection, showing the implication of several DEPs. EBV, known as human herpesvirus 4, was the first human oncovirus to be discovered in Burkitt lymphoma in 1964 [55]. It was found to be strongly associated with nasopharyngeal carcinoma, particularly undifferentiated non-keratinizing carcinomas, which account for all NPC cases in our study, involving the expression of several latent genes, including the Epstein–Barr nuclear antigens (EBNAs) and latent membrane proteins (LMPs) [56]. The EBV infection pathway involves the activation of several proteins by the EBV latent proteins LMP1 and LMP2. This leads to the overexpression of DEPs including PI3K, NFkB, p38 MAPK, STAT1, and RAC1 through the activation of NF-kappa B, MAPK, and JAK-STAT signaling pathways, which plays an important role in cell proliferation and survival [57]. NFKB2, an observed overexpressed DEP activated by LMP1, was found to be involved in the non-canonical inflammatory NF-kB pathway and enriched in most NPC tumors [58]. The dysregulation of the NF-kB pathway has been widely reported in NPC [59]. However, it is still unclear whether the canonical or the non-canonical NF-kB pathway plays a bigger role in NPC’s oncogenesis. Similar to our findings, Tay and colleagues observed an up-regulation of members of the non-canonical arm of NF-kB, including NFKB2 [58]. Furthermore, BCL2, which is involved in preventing cell death (apoptosis) [50], was one of the major oncogenes activated in the EBV infection pathway with the implication of EBV genes such as EBNA3C, BARF-1, and BHRF1. We note that the DEPs mentioned in the EBV infection pathway were identified with 3 or more peptides ranging from 3 to 39. These findings are consistent with the existing literature and indicate that the EBV latent genes, particularly LMP1, might potentially serve as a biomarker for NPC.

By investigating the prognostic significance of the ten most significant DEPs between NPC cases and controls, focusing on clinicopathological characteristics such as stage categories (early versus late) and the TNM stage, we found that PPIA (also known as CyPA) protein intensity was statistically significant across different T stages. This finding indicates that PPIA expression varies significantly with tumor progression, highlighting its potential role as a prognostic biomarker. PPIA has been reported to be involved in the activation of the NF-kappa-B and ERK, JNK, and p38 MAPK pathways, as well as the BCL-2 protein, all of which were found to be involved in NPC [60,61]. In addition, Yang et al. showed that PPIA exhibits significant changes in expression during the carcinogenic process, suggesting its potential role as a biomarker for NPC [62]. Recently, a study connected the CYPA or PPIA protein to EBV by reporting an interaction with LMP1 and its binding to AKT1, leading to the activation of the AKT/mTOR/NF-kB signaling cascade [63].

Apart from the North African population, NPC has been found to be highly prevalent in Southeast Asia, particularly among the Cantonese population. From a molecular perspective, several studies reported the association of certain proteins with NPC risk in the Cantonese population, including CYP2E1, XRCC1, and TRIM26 [64,65,66]. This aligns with our study where TRIM26 was identified as a differentially expressed protein between patients with NPC and healthy individuals.

There were some limitations of the current study. Firstly, the size of the effective cohort contained 41 NPC samples and 22 control samples. Additionally, the healthy control samples used in our study were acquired from data from the ProteomeXchange consortium. Unfortunately, collecting healthy nasopharyngeal tissues in Morocco proved to be impractical. As noted, these samples come from an underserved community and access to tumor tissues is non-trivial. Ultimately, additional follow-up with a larger cohort will be required to identify significant proteins that have either smaller extents of differential expression, or larger extents of biological variation. Secondly, TNM staging information was only available for 22 out of the 41 NPC samples. Furthermore, the silhouette width observed in the k-means clustering analysis was relatively low, although still providing valuable insights. Future work could increase the number of patients and more deeply interrogate the patient groups.

Moving forward, a more comprehensive analysis of a larger cohort of matched tumor–serum samples would facilitate a better understanding of the molecular profile of nasopharyngeal carcinoma for biomarker discovery, particularly in the case versus control and early vs. advanced stage setting. Our future directions will also include a deeper analysis of the interesting DEPs from our findings, the Epstein–Barr virus pathway from a multi-omic perspective, and the male–female comparison to further confirm the remarkable sex disparity in NPC.

## 5. Conclusions

Our research has provided insights into the molecular landscape of nasopharyngeal carcinoma from a proteomic perspective with a focus on differentially expressed proteins across four conditions: the comparison of proteomic clusters in NPC cases, NPC cases versus controls, early versus advanced stages, and males versus females. Through a pathway analysis, we have identified enriched terms and pathways related to the immune response and increased cellular activity leading to uncontrolled cell growth and proliferation. Our findings have demonstrated the involvement of several signaling pathways including PI3K, NF-kB, and p38-MAPK, which are crucial in the initiation and development of NPC. We also revealed the implication of several interesting proteins in NPC carcinogenesis and prognoses. These results improve our understanding of NPC proteomics and its molecular profile while highlighting potential targets that may ultimately help improve early diagnoses and therapeutic intervention in this highly complex disease.

## Figures and Tables

**Table 1:** Differentially expressed proteins observed among all conditions; NPC cluster 1 versus 2, NPC cluster 1 versus 3, NPC cluster 2 versus 3, NPC versus control, male versus female, and early versus advanced stage of NPC.

Condition	Samples	DEPs	Up-Regulated Proteins	Down-Regulated Proteins
NPC cluster 1 vs. 2	41	153	129	24
NPC cluster 1 vs. 3	41	544	468	76
NPC cluster 2 vs. 3	41	475	29	446
NPC vs. control	62	1507	1408	99
Male vs. female	41	59	30	29
Early vs. advanced stage of NPC	22	27	9	17

**Table 2:** The 10 most significant differentially expressed proteins observed in NPC samples across the three cluster comparisons.

DEPs	Cluster Comparisons	Log2FC	*p*-Value
RBBP7	1 vs. 2	−6.76	6.07 × 10^−8^
SRSF10	1 vs. 2	2.75	3.12 × 10^−6^
FKBP4	1 vs. 2	3.81	1.01 × 10^−4^
HCFC1	1 vs. 2	−1.03	3.07 × 10^−4^
MBNL1	1 vs. 2	−2.35	4.1 × 10^−4^
AASDHPPT	1 vs. 2	2.08	4.8 × 10^−4^
GBP5	1 vs. 2	3.65	4.8 × 10^−4^
PSMA5	1 vs. 2	2.18	5.05 × 10^−4^
CNP	1 vs. 2	3.02	6.01 × 10^−4^
ACADVL	1 vs. 2	2.27	9.31 × 10^−4^
HNRNPA2B1	1 vs. 3 and 2 vs. 3	2.89 and −4.02	2.66 × 10^−14^ and 2.17 × 10^−9^
HNRNPK	1 vs. 3	2.95	9.32 × 10^−12^
H2BC21	1 vs. 3 and 2 vs. 3	3.62 and −5.67	9.86 × 10^−12^ and 3.31 × 10^−9^
CPSF6	1 vs. 3 and 2 vs. 3	2.03 and −2.90	1.16 × 10^−11^ and 2.55 × 10^−8^
MACROH2A1	1 vs. 3 and 2 vs. 3	2.49 and −3.67	1.22 × 10^−11^ and 3.64 × 10^−8^
PPIA	1 vs. 3 and 2 vs. 3	1.92 and −2.75	1.41 × 10^−11^ and 1.28 × 10^−7^
RPS3	1 vs. 3	1.89	2.19 × 10^−11^
DDX39B	1 vs. 3 and 2 vs. 3	6.86 and −6.97	5.91 × 10^−11^ and 6.01 × 10^−10^
DAZAP1	1 vs. 3	2.15	6.74 × 10^−11^
UBA1	1 vs. 3 and 2 vs. 3	2.32 and −3.66	6.76 × 10^−11^ and 6.92 × 10^−8^
ILF2	2 vs. 3	−3.25	6.-36 × 10^−9^
HSP90AB1	2 vs. 3	−5.73	7.76 × 10^−8^
RBM39	2 vs. 3	−2.95	1.29 × 10^−7^

**Table 3:** The 10 most significant differentially expressed proteins observed in the NPC compared to control samples’ condition.

DEPs	Log2FC	*p*-Value	adj-*p-*Value
Ribosomal_uL29	3.9609439	2.22 × 10^−16^	8.67 × 10^−15^
MAPK14	7.99893619	2.22 × 10^−16^	8.67 × 10^−15^
LRRC59	4.44564909	2.22 × 10^−16^	8.67 × 10^−15^
ADH7	11.069019	2.22 × 10^−16^	8.67 × 10^−15^
KIF2A	4.01748049	2.22 × 10^−16^	8.67 × 10^−15^
PPIA	−4.298877	2.22 × 10^−16^	8.67 × 10^−15^
HEXB	14.2337803	4.44 × 10^−16^	1.64 × 10^−14^
FAH	6.91773695	4.44 × 10^−16^	1.64 × 10^−14^
CYB5R1	5.68420721	4.44 × 10^−16^	1.64 × 10^−14^
COPS3	3.70358234	4.44 × 10^−16^	1.64 × 10^−14^

## Data Availability

The datasets presented in this article are not readily available as they are part of an ongoing study. However, they will be made accessible upon reasonable request and published in a public repository later on.

## Supplementary Materials

The following supporting information can be downloaded at: https://www.mdpi.com/article/10.3390/cancers16193282/s1, Table S1: LFQ intensities for identified proteins in all samples; Table S2: Differentially expressed proteins of NPC cluster 1 versus 2; Table S3: Differentially expressed proteins of NPC cluster 1 versus 3; Table S4: Differentially expressed proteins of NPC cluster 2 versus 3; S5: Protein cluster results and discussion; S6: Gene Ontology and KEGG pathway figures of the three NPC clusters’ comparison; Table S7: Gene Ontology of NPC cluster 1 versus 2; Table S8: Gene Ontology of NPC cluster 1 versus 3; Table S9: Gene Ontology of NPC cluster 2 versus 3; Table S10: Differentially expressed proteins of patients with early versus advanced stages of NPC; Table S11: Differentially expressed proteins of male versus female patients; Table S12: Differentially expressed proteins of patients with NPC versus controls; S13: The prognostic significance of the 10 most significant DEPs between NPC clusters and between NPC cases and controls; Table S14: KEGG pathways of the three NPC cluster comparisons; Table S15: Gene Ontology pathway analysis of male vs. female patients; Table S16: Gene Ontology pathway analysis of patients with early vs. advanced stage of NPC; Table S17: Gene Ontology pathway analysis of patients with NPC versus controls; Table S18: KEGG pathways of male vs. female patients; Table S19: KEGG pathways of NPC cases versus controls; S20: Discussion of interesting DEPs from our study. References [67,68,69,70,71,72,73,74] are cited in Supplementary Materials.
