# Supplementary material for: A Proteomic Analysis of Nasopharyngeal Carcinoma in a Moroccan Subpopulation
Source: Cancers (Basel). 2024 Sep 26;16(19):3282. doi: 10.3390/cancers16193282 (PMC11476039; doi:10.3390/cancers16193282)
Supplement: Supplementary file 1 [file cancers-16-03282-s001.zip › Supplemental material S13.pdf]

Supplemental material S13

The prognostic significance of the 10 most significant DEPs in NPC clusters 1vs2, 1vs3, and 2vs3, as well as between patients with NPC and healthy individuals on clinicopathological characteristics of NPC was examined. The T-test was utilized to compare protein expression in early versus advanced stages of NPC, while the ANOVA test was conducted to assess protein expression significance in T (Tumor progression) and N (Nodal extension) stages. Evaluation of protein expression in M stage was not included as only one patient out of the 22 NPC cases with TNM information had metastasis at diagnosis. Tukey's test was used to analyze group differences after finding significance in the ANOVA test.

- **Stage categories:**

Cluster 1vs2

Two sample t-test was significant in RBBP7 (p-val = 0.0090)

Box plot representing its LQF intensity in early versus advanced stages of NPC:

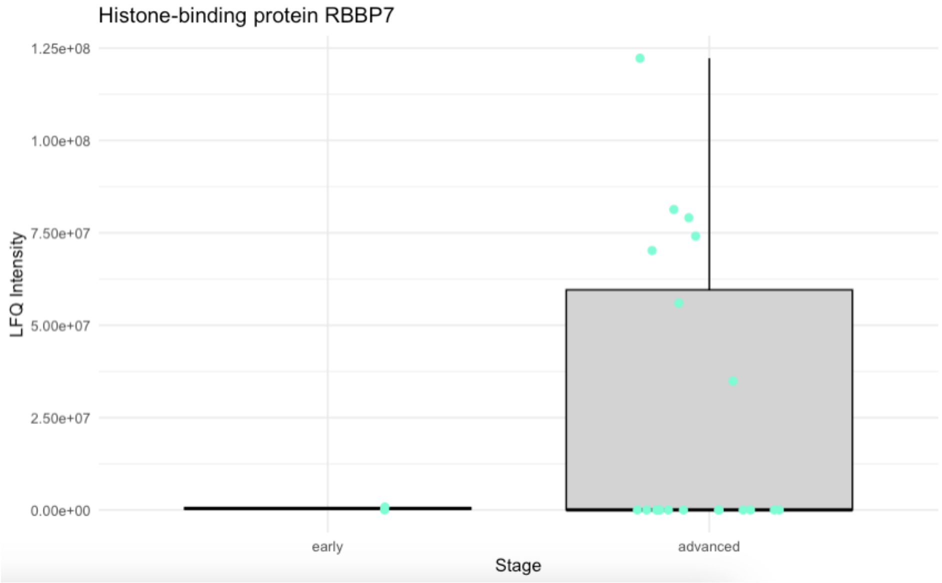

Cluster 1vs3

Two sample t-test was not significant in all DEPs

Cluster 2vs3

Two sample t-test was not significant in all DEPs

NPC samples vs controls

Two sample t-test was not significant in all DEPs

- **T stages:**

Cluster 1vs2

ANOVA test was significant in AASDHPPT (p-val=0.0153) and ACADVL (p-val=0.0707) proteins.

Tukey's test for AASDHPPT: The difference between T2 and T3 as well as T2 and T4 was significant.

```
  Tukey multiple comparisons of means
    95% family-wise confidence level

Fit: aov(formula = LFQ_intensity ~ T_Stage, data = dataT_ADPPT)

$T_Stage
           diff       lwr         upr     p adj
T2-T1   6954617  -2426101 16335334.66 0.1922097
T3-T1  -4639750 -12020459  2740959.45 0.3160026
T4-T1  -2467417 -10126741  5191907.50 0.7995496
T3-T2 -11594367 -20749010 -2439723.29 0.0104958
T4-T2  -9422033 -18802751   -41315.34 0.0487779
T4-T3   2172333  -5208376  9553042.78 0.8386737
```

Box plot representing its LQF intensity across T stages of NPC:

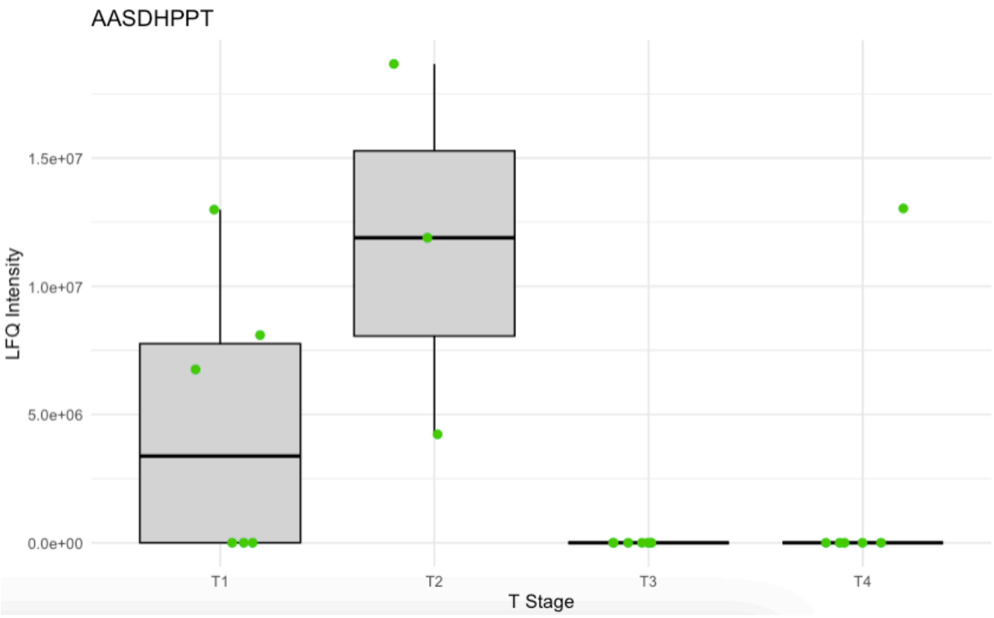

Tukey's test for ACADVL: The difference between T1 and T2 was significant.

Box plot representing its LQF intensity across T stages of NPC:

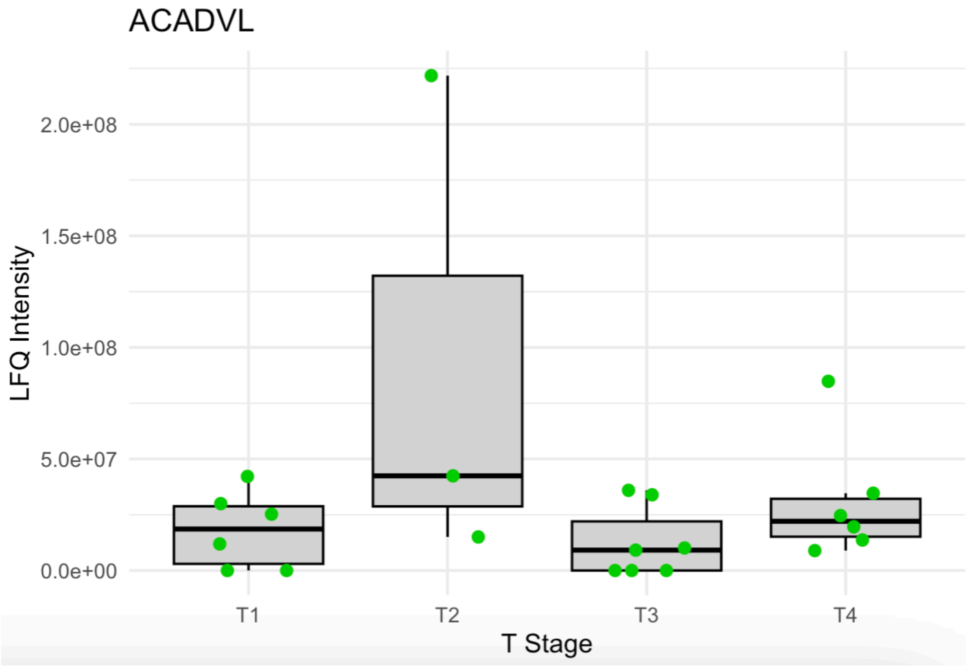

Cluster 1vs3

Two sample t-test was not significant in all DEPs

Cluster 2vs3

Two sample t-test was not significant in all DEPs

NPC samples vs controls

ANOVA test was significant in PPIA protein only (p-val=0.0487)

Tukey's test for PPIA protein: The difference between T2 and T3 was significant.

```
    95% family-wise confidence level

Fit: aov(formula = LFQ_intensity ~ T_Stage, data = dataT_CYB5R1)

$T_Stage
           diff       lwr      upr     p adj
T2-T1  26894833 -19678641 73468307 0.3865784
T3-T1 -17850833 -54494647 18792980 0.5289346
T4-T1   1213000 -36814082 39240082 0.9997270
T3-T2 -44745667 -90196723   705390 0.0545348
T4-T2 -25681833 -72255307 20891641 0.4256059
T4-T3  19063833 -17579980 55707647 0.4746595
```

Box plot representing its LQF intensity across T stages of NPC:

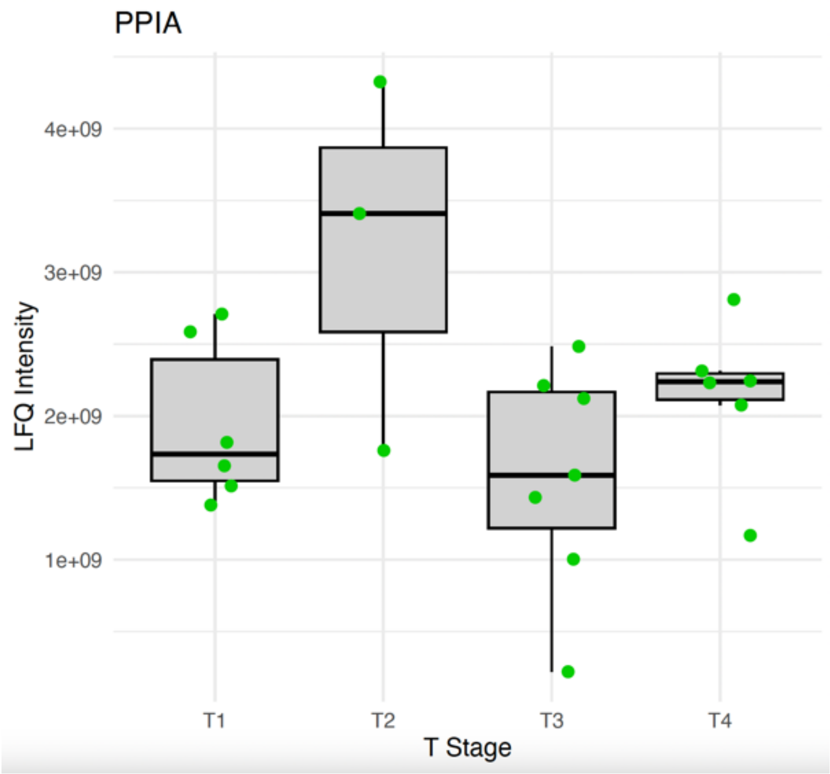

- **N stages**

Cluster 1vs2

ANOVA test was not significant in all DEPs.

Cluster 1vs3

ANOVA test was significant in HNRNPK (p-val= 0.0206) and DAZAP1 (p-val= 0.0444) proteins.

Tukey's test for HNRNPK protein: The difference between Nx-N0, Nx-N1, and Nx-N2 was significant.

```
  Tukey multiple comparisons of means
    95% family-wise confidence level

Fit: aov(formula = LFQ_intensity ~ N_Stage, data = dataN_HNRPK)

$N_Stage
             diff         lwr        upr     p adj
N0-Nx -1603820000 -2873195521 -334444479 0.0099085
N1-Nx -1369864000 -2574099356 -165628644 0.0217306
N2-Nx -1314336364 -2462529724 -166143003 0.0207905
N3-Nx -1184030000 -2530406059  162346059 0.1000541
N1-N0   233956000  -568867571 1036779571 0.8979417
N2-N0   289483636  -426540725 1005507998 0.7347055
N3-N0   419790000  -583739463 1423319463 0.7105105
N2-N1    55527636  -537396865  648452138 0.9984069
N3-N1   185834000  -733915946 1105583946 0.9707393
N3-N2   130306364  -714741069  975353796 0.9891719
```

Box plot representing its LQF intensity across T stages of NPC:

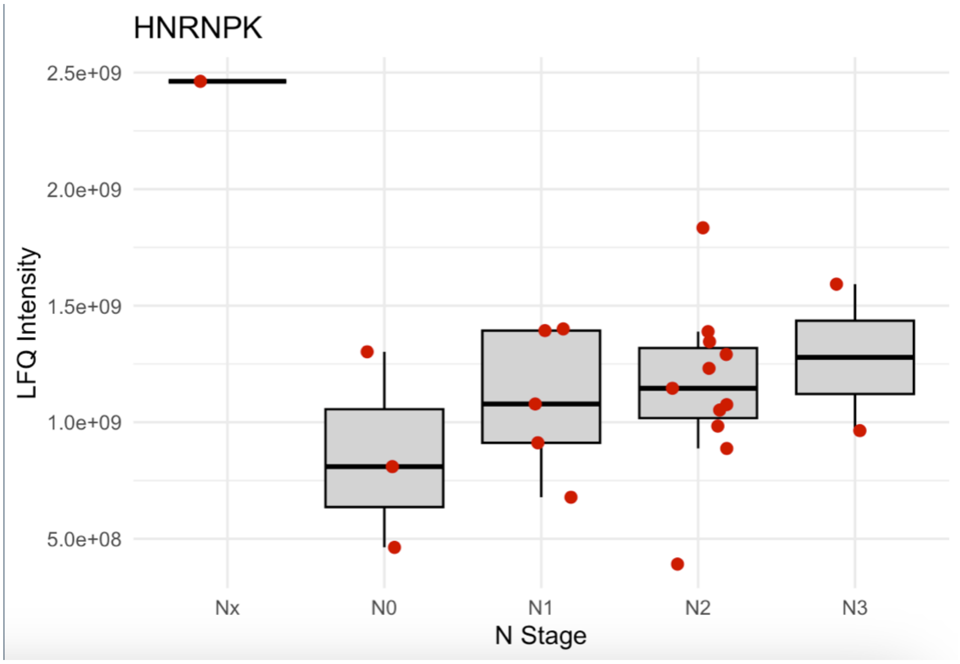

Tukey's test for DAZAP1 protein: The difference between Nx-N0 and Nx-N1 was significant.

$N_Stage

	diff	lwr	upr	p adj
N0-Nx	-136832000	-262562818	-11101182	0.0293976
N1-Nx	-108074200	-227352927	11204527	0.0861694
N2-Nx	-99973909	-213701713	13753895	0.1002528
N3-Nx	-73589500	-206947171	59768171	0.4715485
N1-N0	28757800	-50761351	108276951	0.8040351
N2-N0	36858091	-34063655	107779837	0.5279939
N3-N0	63242500	-36156439	162641439	0.3369295
N2-N1	8100291	-50628494	66829076	0.9928960
N3-N1	34484700	-56615933	125585333	0.7774682
N3-N2	26384409	-57316988	110085806	0.8694537

Box plot representing its LQF intensity across T stages of NPC:

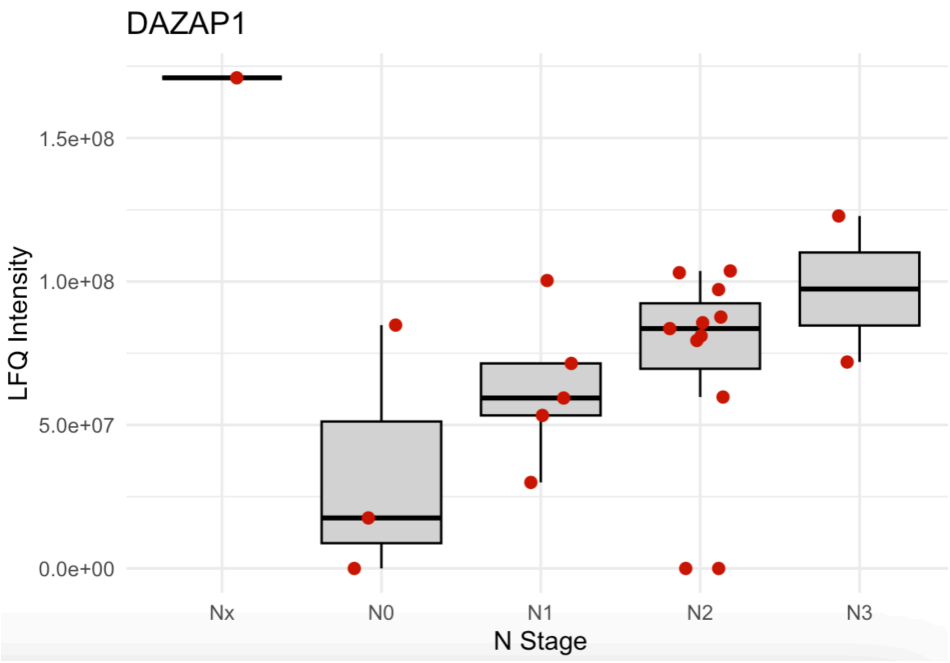

Cluster 2vs3

ANOVA test was not significant in all DEPs.

NPC samples vs controls

ANOVA test was not significant in all DEPs.
