# Supplementary material for: A Proteomic Analysis of Nasopharyngeal Carcinoma in a Moroccan Subpopulation
Source: Cancers (Basel). 2024 Sep 26;16(19):3282. doi: 10.3390/cancers16193282 (PMC11476039; doi:10.3390/cancers16193282)
Supplement: Supplementary file 1 [file cancers-16-03282-s001.zip › Supplemental material S20.pdf]

Supplemental material S20

Discussion

-Early versus Advanced stages of NPC condition:

The vesicle amine transporter 1 (VAT1) was another up-regulated DEP of the immune response cluster, found to be overexpressed in hepatocellular carcinoma and glioblastomas with involvement in migration and association with shorter OS [1,2]. This differentially expressed protein might serve as a diagnostic marker with its involvement in disease progression.

MARC2 also known as Mitochondrial amidoxime-reducing component 2, was among downregulated differentially expressed proteins. MARC2 has been found to facilitate immune escape and was associated with immunosuppression of hepatocellular carcinoma [3], making it a protein of interest.

-NPC versus controls condition:

Histone 3 DEPs (H3C1 to H3C15) were also highly enriched and upregulated in this study, although very few studies reported their direct implication in carcinogenesis. In gastric cancer, Rashid et al. reported an overexpression of the H3C14 histone gene mediated through the EGFR-FOXC1 axis [4]. H3 proteins are known to regulate DNA structure and gene expression within the cell.

References

1. Mertsch, S.; Becker, M.; Lichota, A.; Paulus, W.; Senner, V. Vesicle Amine Transport Protein-1 (VAT-1) Is Upregulated in Glioblastomas and Promotes Migration. *Neuropathol Appl Neurobiol* **2009**, *35*, 342–352, doi:10.1111/j.1365-2990.2009.00993.x.
2. Heng, W.; Wei, F.; Li, W.; Li, Q.; Xiong, D.-L.; Ma, Y.-Y.; Zhang, D.-H. Expression of VAT1 in Hepatocellular Carcinoma and Its Clinical Significance. *Neoplasma* **2021**, *68*, 416–422, doi:10.4149/neo_2020_201008N1061.
3. Wu, D.; Liu, L. Downregulation of MARC2 Promotes Immune Escape and Is Associated With Immunosuppression of Hepatocellular Carcinoma. *Front. Genet.* **2022**, *12*, doi:10.3389/fgene.2021.790093.
4. Rashid, M.; Shah, S.G.; Verma, T.; Chaudhary, N.; Rauniyar, S.; Patel, V.B.; Gera, P.B.; Smoot, D.; Ashaktorab, H.; Dalal, S.N.; et al. Tumor-Specific Overexpression of Histone Gene, H3C14 in Gastric Cancer Is Mediated through EGFR-FOXC1 Axis. *Biochim Biophys Acta Gene Regul Mech* **2021**, *1864*, 194703, doi:10.1016/j.bbagrm.2021.194703.
