# Supplementary material for: A Proteomic Analysis of Nasopharyngeal Carcinoma in a Moroccan Subpopulation
Source: Cancers (Basel). 2024 Sep 26;16(19):3282. doi: 10.3390/cancers16193282 (PMC11476039; doi:10.3390/cancers16193282)
Supplement: Supplementary file 1 [file cancers-16-03282-s001.zip › Supplemental material S5.pdf]

Supplemental material S5

Protein clusters

Results

To define the most likely clusters, we performed a k-means clustering analysis across a range of k from 1-6. The silhouette scores suggested the most probable number of clusters was 3 (k=3 had a silhouette score of 0.093). The first cluster contained 29 NPC samples, while the second and third contained 2 and 10 NPC samples, respectively.

After identifying which patients belonged in which cluster, we then attempted to identify the proteins that differentiated amongst the clusters. The comparison between cluster 1 (n=29) and cluster 3 (n=10), being the more substantial comparison, showed 544 DEPs, with 468 up-regulated (fold change ≥1) and 76 down-regulated proteins (fold change ≤1). The other comparisons involving cluster 2 (n=2) were also retained to provide a more comprehensive understanding of the data. A total of 153 differentially expressed proteins, with 129 up-regulated and 24 down-regulated proteins were observed between clusters 1 and 2, while 475 DEPs (29 up-regulated and 446 down-regulated) were identified between cluster 2 and cluster 3. The 10 most significant differentially expressed proteins (DEPs) across the NPC patient groups are found in Table 2. Among these differentially expressed proteins, the expression of HNRNPK and DAZAP1 proteins, identified between clusters 1 and 3, showed statistical significance across N stages (Nx-N0, Nx-N1, Nx-N2). the expression of RBBP7, a protein identified in NPC cluster 1 versus 2, showed statistical significance between patients with early and late stages of NPC. AASDHPPT and ACADVL are other DEPs observed between NPC clusters 1 and 2 that were found to be statistically significant across all T stages of NPC, particularly between T2-T3 and T2-T4 for AASDHPPT and between T1-T2 and T2-T3 for ACADVL.

Gene ontology (GO) enrichment analysis was conducted to obtain biological insights into three categories: cellular component (CC), biological process (BP), and molecular function (MF). The significantly enriched terms of each category are presented in **supplemental materials S9-S12**. The analysis of inter-cluster differences between clusters 1 and 3, revealed BP-enriched terms in “cytoplasmic translation”, “translation”, “telomere organization”, “nucleosome assembly”, and “chromatin organization”. CC-enriched terms were involved in “extracellular exosome”, “cytosol”, “cytosolic ribosome”, “focal adhesion”, and “nucleus”. The DEPs in molecular function revealed enriched terms related to “RNA binding”, “protein binding”, “cadherin binding”, “structural constituent of chromatin”, and “structural constituent of ribosome”. Regarding cluster 1 versus 2, the main BP enriched terms were involved in “mRNA splicing, via

spliceosome", "negative regulation of mRNA splicing, via spliceosome", "chaperone-mediated protein complex assembly", protein stabilization", and "protein refolding". The main enriched CC terms were related to "extracellular exosome", "nucleoplasm", "cytosol", "cytoplasm", and "nucleus". MF showed enriched terms in "RNA binding", "ATP-dependent protein folding chaperone", "protein binding", unfolded protein binding", and "protein folding chaperone". The main enriched terms of biological process between clusters 2 and 3 were involved in "cytoplasmic translation", "telomere organization", "nucleosome assembly", "translation", and "chromatin organization". The main CC terms were related to "extracellular exosome", "cytosol", "nucleoplasm", "nucleus", "ribonucleoprotein complex", and "focal adhesion". The main MF terms were concerning "RNA binding", "protein binding", "structural constituent of chromatin", "cadherin binding", and mRNA binding".

An examination of Kyoto Encyclopedia of Genes and Genomes Pathway (KEGG) enrichment analysis results (Figures S4-S6 in Supplemental material S9) revealed pathway enrichment in NPC cluster 1 versus 3 were related to "Ribosome", "Coronavirus disease_COVID_19", "Systemic lupus erythematosus", "Neutrophil extracellular trap formation", and "Alcoholism". Regarding NPC cluster 1 versus 2, the main pathways were involved in "Proteasome", "Antigen processing and presentation", "Valine, leucine, and isoleucine degradation", "Metabolic pathways", and "Glycolysis/Gluconeogenesis". The enriched KEGG pathways between clusters 2 and 3 were related to "Systemic lupus erythematosus", "Ribosome", "Coronavirus disease_COVID_19", "Neutrophil extracellular trap formation", and "Alcoholism".

Discussion

Using our NPC dataset, we attempted to identify and characterize proteomically defined subtypes. Pathway analysis was performed on the identified DEPs across the three cluster comparisons to elucidate their biological significance in NPC. Although the comparison between clusters 1 and 3 was more substantial given their higher sample size, we retained the other comparisons providing valuable insights. The gene ontology analysis for cluster 1 compared to cluster 2 revealed significant enrichment in biological processes related to RNA/protein processing and stabilization, as well as immune response and telomere maintenance. Similar enriched pathways were observed in the differentially expressed proteins (DEPs) for cluster 1 vs. 3 and cluster 2 vs. 3, in addition to gene expression and translation processes, indicating increased cellular activity and immune response. Functional annotation clustering analysis, combining BP, CC, and MF from Gene Ontology and KEGG pathways results, was conducted to confirm these findings further. In the comparison between clusters 1 and 2, the most significant cluster was associated with protein maintenance, stress response, and cellular defense (including immune response and regulation of apoptosis and cell survival). When comparing clusters 1 and 3, the most significant functional annotation cluster was related to the

translation process and ribosomes. Notably, the most significant cluster between clusters 2 and 3 was also related to the translation process and ribosomes, with both having cluster 3 in common.
In both cluster 1 compared to 3 and cluster 2 compared to 3, 7 out of the 10 most significant differentially expressed proteins were the same. All of these proteins were upregulated in cluster 1vs 3 and downregulated in cluster 2vs 3. In terms of prognostic significance, the expression of RB binding protein 7, a downregulated DEP in cluster 1 versus 2, was found to be significant between patients with early and advanced stages of NPC. RBBP7, implicated in protein binding, chromatin regulation, and negative regulation of the transcription process, has been found to be involved in several cancer types, driven by the many epigenetic alterations leading to its upregulation in cancer cells [1]. However, similar to our results, numerous studies concluded a tumor suppressor role for RBBP4/7 [2]. It is still an understudied protein that needs further attention. The expression of both AASDHPPT and ACADVL upregulated protein was found to be significant between clusters 1 and 2 across T stages of NPC samples. These two DEPs, playing a role in metabolic pathways, were previously reported to be involved in cancer [3,4]. HNRNPK and DAZAP1 upregulated proteins were both found to be significantly expressed across N stages between clusters 1 and 3. Several studies have shown the implication of the HNRNPK protein in cancer by interacting with other proteins and signaling pathways such as the WNT pathway [5]. Chung et al. found that HNRNPK's high expression was correlated with the overexpression of MMP12 in NPC tissues, suggesting a potential therapeutic role for this protein [6]. Although DAZAP1 was less reported, it was shown to be involved in cell proliferation with its important role in m-RNA splicing activation [7]. Deng et al. found that the overexpression of DAZAP1 in hepatic carcinoma was a predictor of poor prognosis [8]. These proteins, found to have prognostic significance in our study, might serve as novel markers, specifically in the early stages of NPC.

References:

1. Cai, L.; Liu, B.; Cao, Y.; Sun, T.; Li, Y. Unveiling the Molecular Structure and Role of RBBP4/7: Implications for Epigenetic Regulation and Cancer Research. *Front. Mol. Biosci.* **2023**, *10*, doi:10.3389/fmolb.2023.1276612.
2. Kong, L.; Yu, X.-P.; Bai, X.-H.; Zhang, W.-F.; Zhang, Y.; Zhao, W.-M.; Jia, J.-H.; Tang, W.; Zhou, Y.-B.; Liu, C. RbAp48 Is a Critical Mediator Controlling the Transforming Activity of Human Papillomavirus Type 16 in Cervical Cancer. *J Biol Chem* **2007**, *282*, 26381–26391, doi:10.1074/jbc.M702195200.
3. Wheeler, L.J.; Watson, Z.L.; Qamar, L.; Yamamoto, T.M.; Sawyer, B.T.; Sullivan, K.D.; Khanal, S.; Joshi, M.; Ferchaud-Roucher, V.; Smith, H.; et al. Multi-Omic Approaches Identify Metabolic and Autophagy Regulators Important in Ovarian Cancer Dissemination. *iScience* **2019**, *19*, 474–491, doi:10.1016/j.isci.2019.07.049.
4. Wang, P.; Zheng, H.; Zhang, J.; Wang, Y.; Liu, P.; Xuan, X.; Li, Q.; Du, Y. Identification of Key Gene Modules and Genes in Colorectal Cancer by Co-Expression Analysis Weighted Gene Co-Expression Network Analysis. *Biosci Rep* **2020**, *40*, BSR20202044, doi:10.1042/BSR20202044.
5. Zhu, L.; Tian, Q.; Gao, H.; Wu, K.; Wang, B.; Ge, G.; Jiang, S.; Wang, K.; Zhou, C.; He, J.; et al. PROX1 Promotes Breast Cancer Invasion and Metastasis through WNT/β-Catenin Pathway via Interacting with hnRNPK. *Int J Biol Sci* **2022**, *18*, 2032–2046, doi:10.7150/ijbs.68960.

6. Chung, I.-C.; Chen, L.-C.; Chung, A.-K.; Chao, M.; Huang, H.-Y.; Hsueh, C.; Tsang, N.-M.; Chang, K.-P.; Liang, Y.; Li, H.-P.; et al. Matrix Metalloproteinase 12 Is Induced by Heterogeneous Nuclear Ribonucleoprotein K and Promotes Migration and Invasion in Nasopharyngeal Carcinoma. *BMC Cancer* **2014**, *14*, 348, doi:10.1186/1471-2407-14-348.
7. Choudhury, R.; Roy, S.G.; Tsai, Y.S.; Tripathy, A.; Graves, L.M.; Wang, Z. The Splicing Activator DAZAP1 Integrates Splicing Control into MEK/Erk-Regulated Cell Proliferation and Migration. *Nat Commun* **2014**, *5*, 3078, doi:10.1038/ncomms4078.
8. Deng, J.J.; Li, G.P.; Lu, W.; Yan, Z.; Wang, Y. DAZAP1 Overexpression Promotes Growth of HCC Cell Lines: A Primary Study Using CEUS. *Clin Transl Oncol* **2022**, *24*, 1168–1176, doi:10.1007/s12094-021-02758-8.
