# Supplementary material for: A Proteomic Analysis of Nasopharyngeal Carcinoma in a Moroccan Subpopulation
Source: Cancers (Basel). 2024 Sep 26;16(19):3282. doi: 10.3390/cancers16193282 (PMC11476039; doi:10.3390/cancers16193282)
Supplement: Supplementary file 1 [file cancers-16-03282-s001.zip › Supplemental material S6 .pdf]

Supplemental material S6

The significantly enriched terms of Gene Ontology categories (BP, CC, and MF) and KEGG pathways identified in cluster 1vs2, cluster 1vs3, and cluster 2vs3 are as follows:

Gene Ontology:

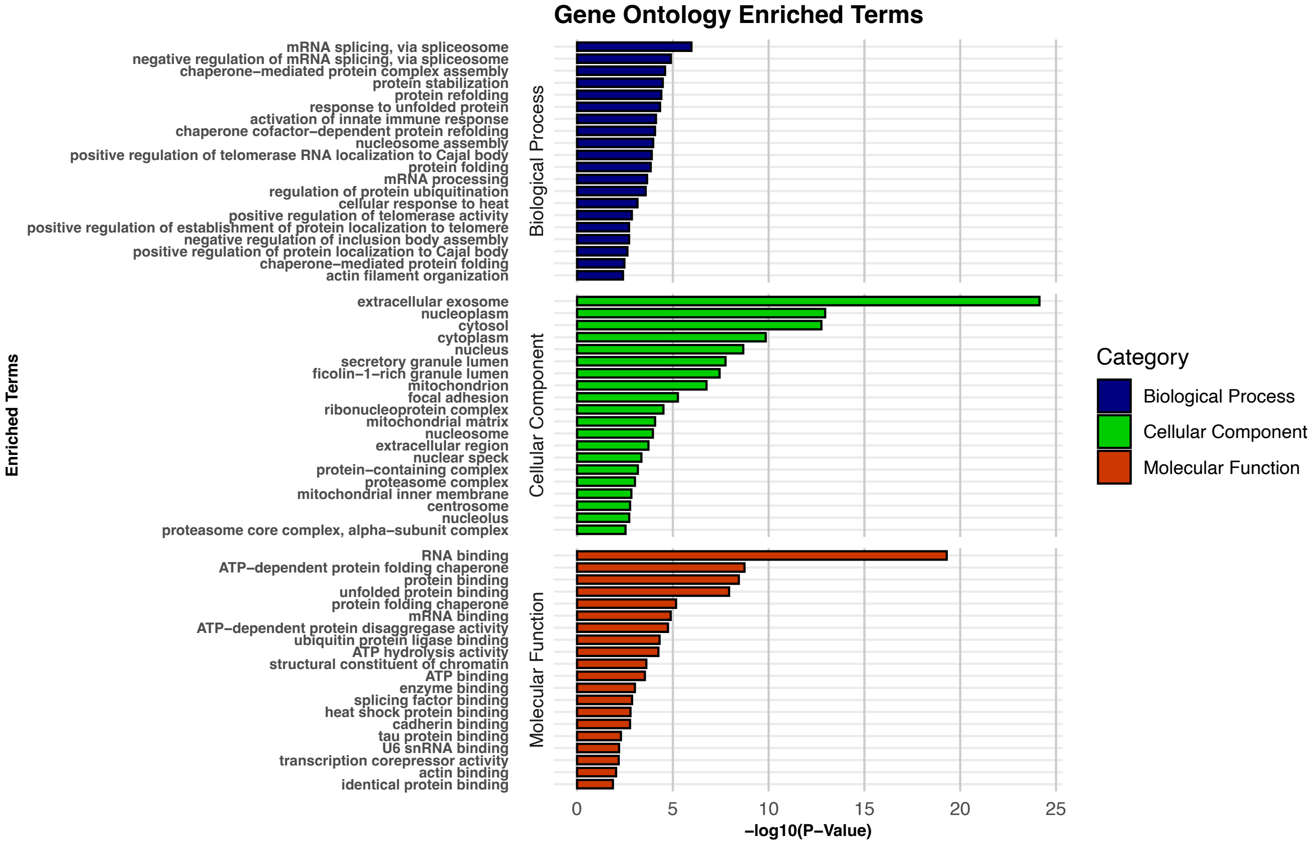

Figure S1: Gene Ontology enriched terms in cluster 1vs2

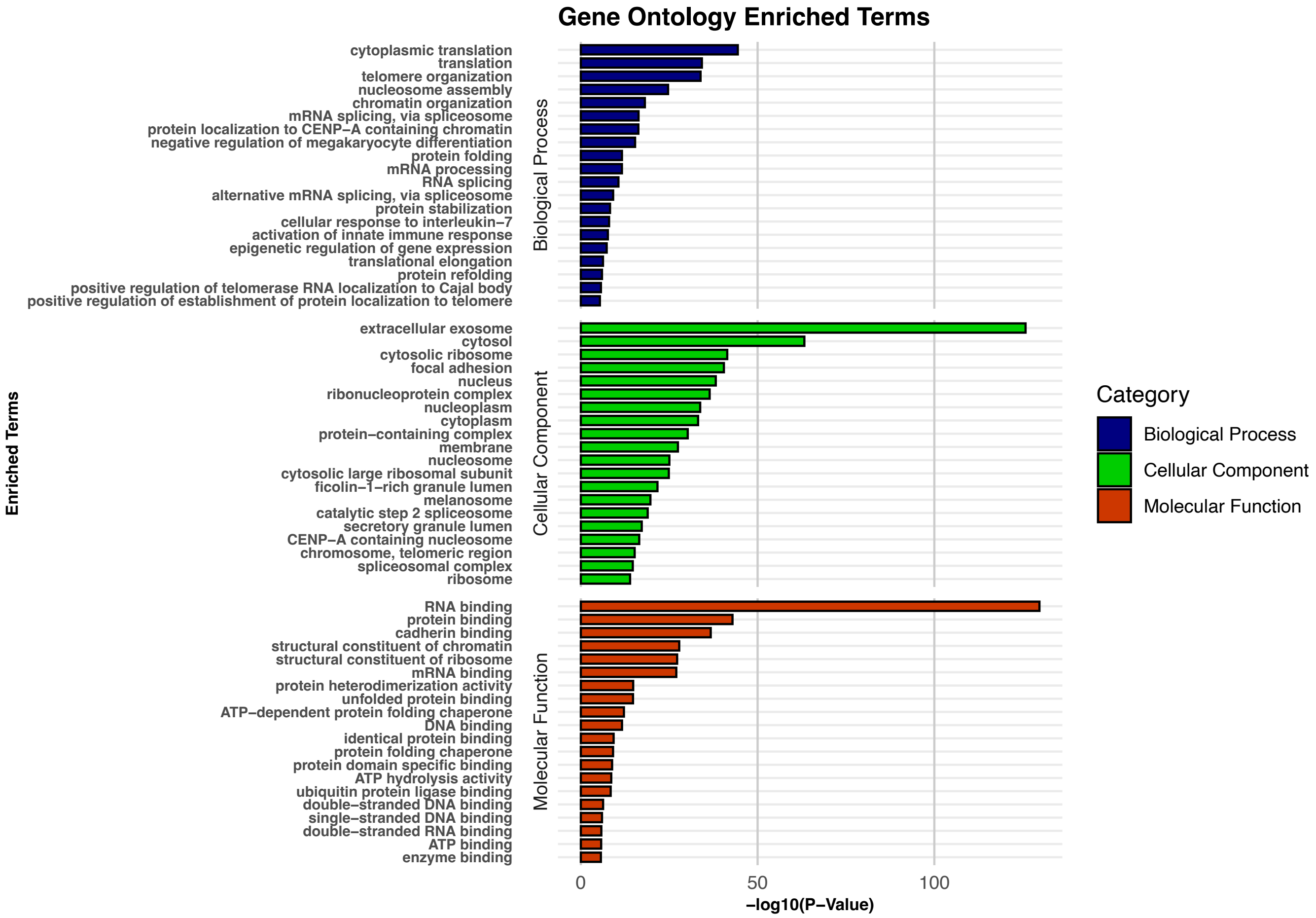

Figure S2: Gene Ontology enriched terms in cluster 1vs3

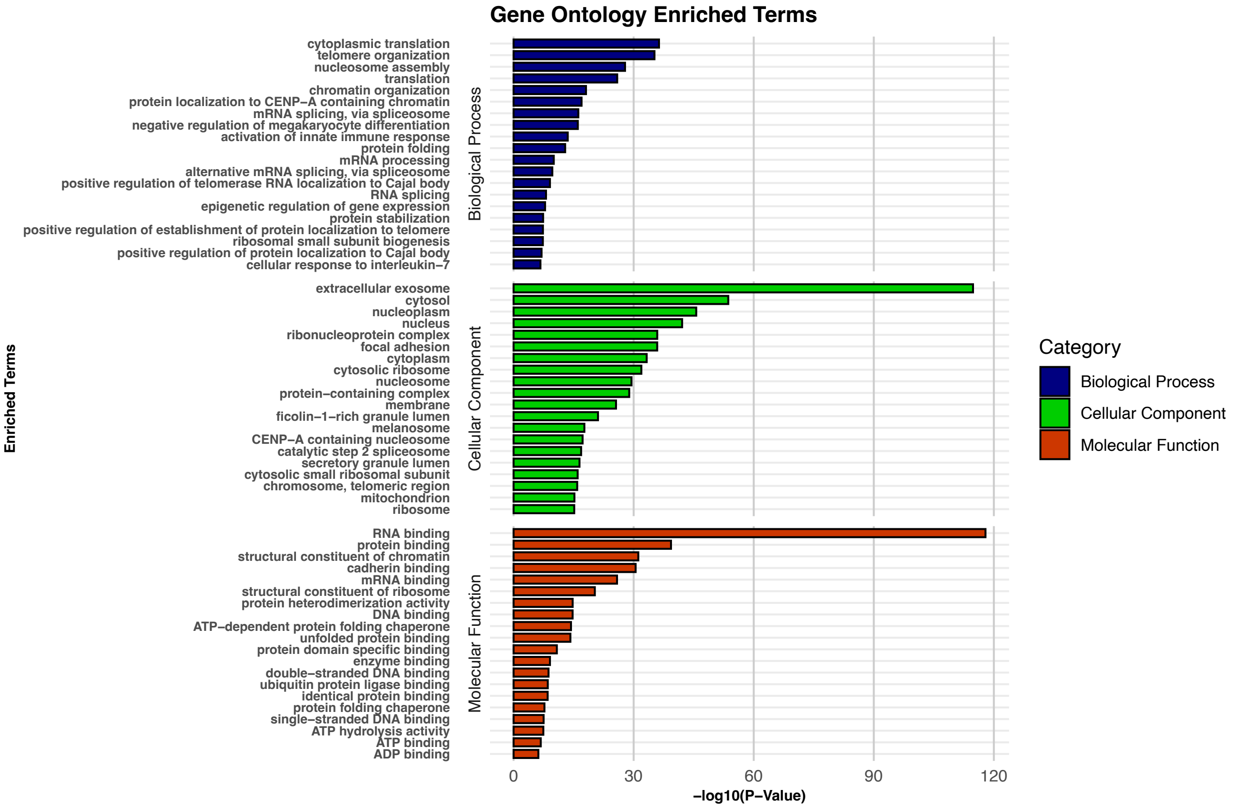

Figure S3: Gene Ontology enriched terms in cluster 2vs3

KEGG pathways:

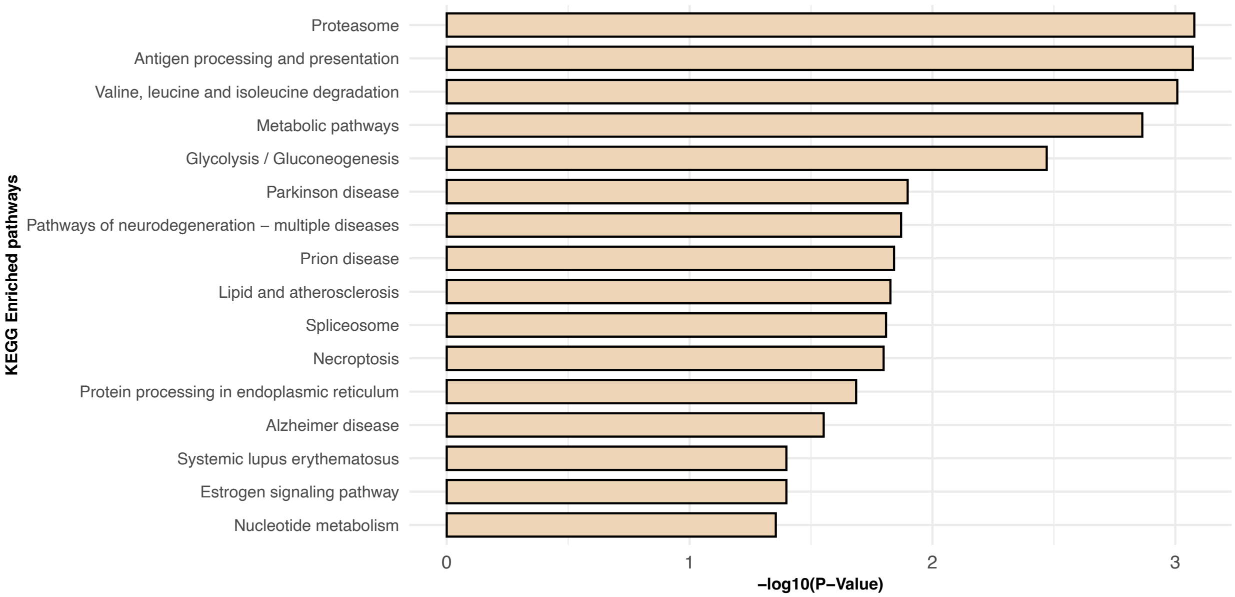

Figure S4: KEGG enriched pathways in cluster 1vs2

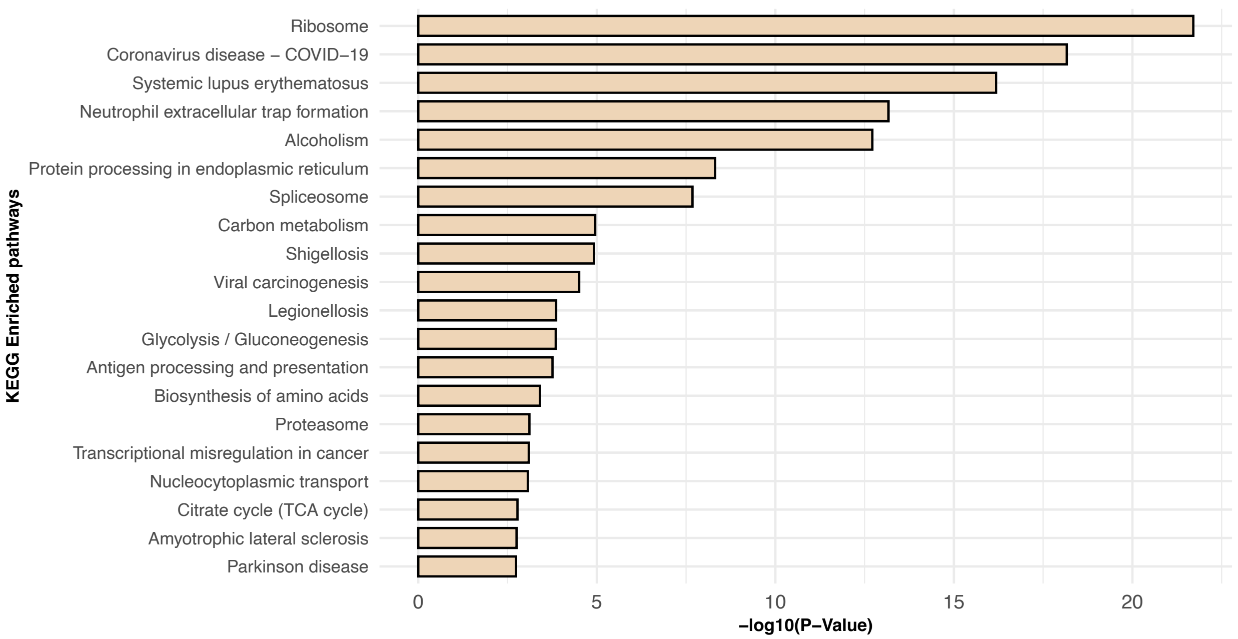

Figure S5: KEGG enriched pathways in cluster 1vs3

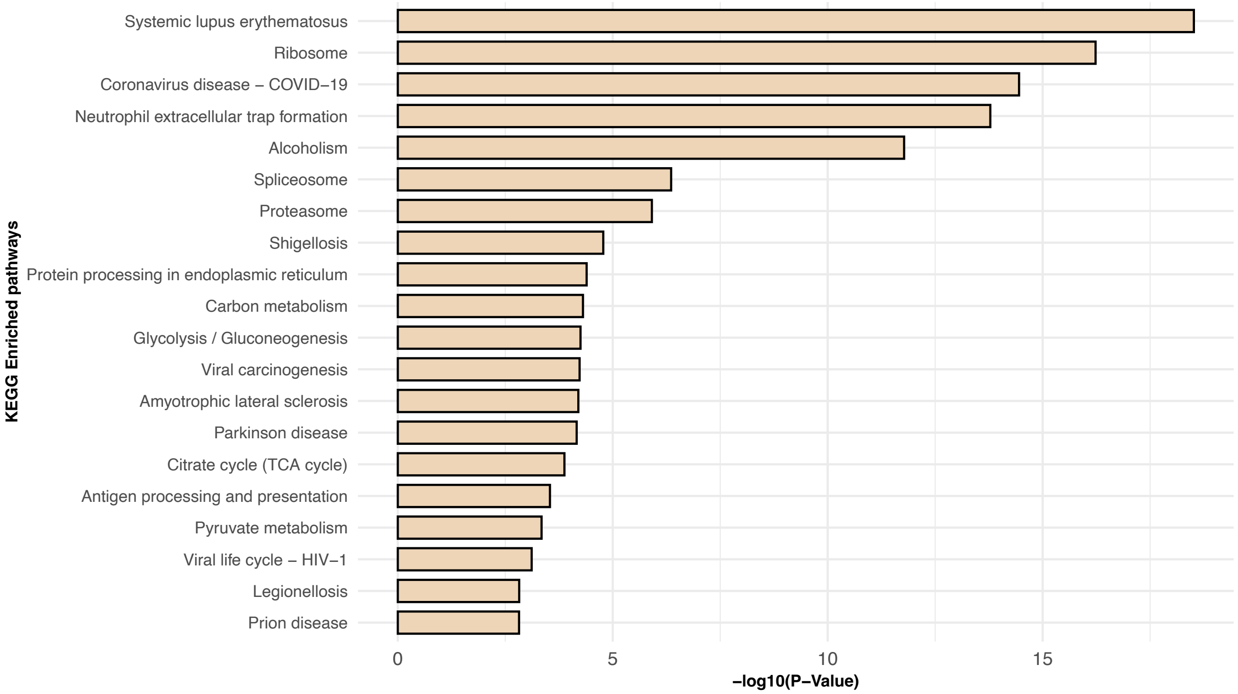

Figure S6: KEGG enriched pathways in cluster 2vs3
